# Genetic risk of depression is different in subgroups of dietary ratio of tryptophan to large neutral amino acids

**DOI:** 10.1038/s41598-023-31495-x

**Published:** 2023-03-27

**Authors:** Bence Bruncsics, Gabor Hullam, Bence Bolgar, Peter Petschner, Andras Millinghoffer, Kinga Gecse, Nora Eszlari, Xenia Gonda, Debra J. Jones, Sorrel T. Burden, Peter Antal, Bill Deakin, Gyorgy Bagdy, Gabriella Juhasz

**Affiliations:** 1 Department of Measurement and Information Systems, Budapest University of Technology and Economics, Muegyetem Rkp. 3., 1111 Budapest, Hungary; 2 Department of Pharmacodynamics, Faculty of Pharmacy, Semmelweis University, Nagyvarad ter 4., 1089 Budapest, Hungary; 3 NAP3.0-SE Neuropsychopharmacology Research Group, Hungarian Brain Research Program, Semmelweis University, Budapest, Hungary; 4 Bioinformatics Center, Institute for Chemical Research, Kyoto University, Gokasho, Uji, Kyoto, Japan; 5 SE-NAP2 Genetic Brain Imaging Migraine Research Group, Hungarian Brain Research Program, Semmelweis University, Budapest, Hungary; 6 Department of Psychiatry and Psychotherapy, Faculty of Medicine, Semmelweis University, Budapest, Hungary; 7 School of Health Sciences, University of Manchester, Manchester, UK; 8 Division of Neuroscience and Experimental Psychology, School of Biological Sciences, Faculty of Biology, Medicine and Health, University of Manchester, Manchester Academic Health Science Centre, Manchester, UK

**Keywords:** Genetics research, Depression, Lifestyle modification, Nutrition

## Abstract

Manipulation of intake of serotonin precursor tryptophan has been exploited to rapidly induce and alleviate depression symptoms. While studies show that this latter effect is dependent on genetic vulnerability to depression, the effect of habitual tryptophan intake in the context of predisposing genetic factors has not been explored. Our aim was to investigate the effect of habitual tryptophan intake on mood symptoms and to determine the effect of risk variants on depression in those with high and low tryptophan intake in the whole genome and specifically in serotonin and kynurenine pathways. 63,277 individuals in the UK Biobank with data on depressive symptoms and tryptophan intake were included. We compared two subpopulations defined by their habitual diet of a low versus a high ratio of tryptophan to other large amino acids (TLR). A modest protective effect of high dietary TLR against depression was found. *NPBWR1* among serotonin genes and *POLI* in kynurenine pathway genes were significantly associated with depression in the low but not in the high TLR group. Pathway-level analyses identified significant associations for both serotonin and kynurenine pathways only in the low TLR group. In addition, significant association was found in the low TLR group between depressive symptoms and biological process related to adult neurogenesis. Our findings demonstrate a markedly distinct genetic risk profile for depression in groups with low and high dietary TLR, with association with serotonin and kynurenine pathway variants only in case of habitual food intake leading to low TLR. Our results confirm the relevance of the serotonin hypothesis in understanding the neurobiological background of depression and highlight the importance of understanding its differential role in the context of environmental variables such as complexity of diet in influencing mental health, pointing towards emerging possibilities of personalised prevention and intervention in mood disorders in those who are genetically vulnerable.

## Introduction

It is increasingly recognised that a great part of somatic, and possibly some mental illnesses are related to lifestyle and nutritional factors, therefore, consideration of nutrient intake with an impact on nervous function in large-scale genetic analyses may hold the promise of revealing important etiological mechanisms. These mechanisms may be obscured in the absence of taking into account such interacting factors, which may also play a central role in behavioural and lifestyle interventions as well as personalised treatment or even prevention. The monoamine theory proposed that low monoamine neurotransmission is one of the main pathophysiological processes in the development of depression. This is based on early evidence that acute depletion of the central nervous system’s monoamine reserve, by reserpine administration, induces depressive symptoms, and drugs that treat depression increase serotonin, norepinephrine, and/or dopamine neurotransmission. This evidence resulted in a shift of the main focus to serotonin^1,2^, a key player in controlling emotion, appetite, sleep, pain, and response to the surrounding environment, thus contributing to depressive symptoms in various ways^3,4^. Although, the extensive research over the past couple of decades provided evidence that serotonin neurotransmission alone cannot explain the pathophysiology of such a heterogeneous disorder^5,6^. Both quantitative alterations, such as availability of the serotonin precursor tryptophan, and qualitative modifications, such as variations in the signal transduction mechanisms of the serotonin system may be relevant in the pathophysiology of depression by modulating brain function in vulnerable individuals^7,8^.

Serotonin is synthesized from the essential amino acid tryptophan, with its plasma concentration dependent on dietary intake. As serotonin cannot cross the blood–brain barrier, central nervous system neurotransmission relies on serotonin synthesis in the cells of the raphe nuclei. Tryptophan crosses into the brain via a transport protein complex (large amino transporter, LAT1), which carries all large neutral amino acids (LNAA) across the blood–brain barrier and is regulated by the availability of tryptophan and other LNAAs competing for the transporter. Therefore, manipulation of dietary tryptophan content by depletion or supplementation leads to changes in brain serotonin concentrations^9–11^.

Acute tryptophan depletion (ATD) induces rapid reduction in serotonin brain synthesis, triggering a brief relapse in remitted depressive patients, especially in those treated with serotonergic antidepressants. Remarkably, healthy volunteers, but only those with a family history of depression, also experience mood lowering effects after ATD, suggesting the role of genetic vulnerability to depression in the response to tryptophan depletion^12,13^.

In contrast, serotonin function can be enhanced by raising plasma tryptophan/LNAA ratio through tryptophan-rich protein administration or a high-carbohydrate and low-protein diet, contributing to improved stress adaptation in stress-prone subjects, as indicated by a lower cortisol response and reduced depressive symptoms after ingestion of alpha-lactalbumin compared to a low tryptophan control protein^14,15^. Furthermore, chronic tryptophan supplementation can induce positive emotional bias, similar to the effect of serotonergic antidepressants in healthy females but not male volunteers^16^. Optimal tryptophan supplementation may thus serve as a preventive strategy in depression because of its beneficial effect on mood and cognition both in healthy volunteers and subjects vulnerable to depression^17–19^. However, to employ a targeted preventive public health strategy, the effect of habitual tryptophan intake should be explored in light of the genetic risk factors that predispose to low mood in case of low dietary tryptophan/LNAA ratio (TLR).

Despite the broad range of experimental studies, the relationship between habitual dietary tryptophan intake and depression is underexplored. Dietary tryptophan intake was inversely correlated with self-reported depression in a US population-based study^20^ and similarly with depressive symptoms in young Japanese women, with a trend in their mothers^21^, but no association was found in a male-only study^22^.

Regarding genetic vulnerability to depression in the face of variable tryptophan intake our knowledge is even more limited. The genetic factors modulating the effect of tryptophan intake manipulation are largely unknown with the exception of some serotonergic candidate variants with conflicting results^23,24^. Although large-scale GWAS provided evidence that risk variants of depression represent synaptic organization and plasticity influencing behaviour and cognition, they failed to replicate previously investigated candidate genes, including serotonin ones^25,26^. However, as dietary TLR influences the metabolic pathway of serotonin synthesis and thus serotonergic signalling, we hypothesized that the serotonin genetic pathway will have a more significant association with depressive symptoms in those whose tryptophan intake is low. We also focused on the kynurenine genetic pathway, as only a small fraction of the plasma tryptophan metabolises to serotonin and the majority uses the kynurenine shunt to transform into cytoprotective (kynurenic acid) or cytotoxic (3-hydroxy-kynurenine and quinolinic acid) compounds^27^. The majority of brain kynurenine originates from the periphery and uses the same LAT1 transporter as tryptophan and LNAAs to cross the blood–brain barrier. Accumulating evidence suggests that stress and peripheral inflammation stimulate the kynurenine pathway by activating the indoleamine 2,3-dioxygenase (IDO) transforming tryptophan into kynurenine. The increased kynurenine/tryptophan ratio may lead to depression by lowering plasma tryptophan concentration and thus interfering with serotonin synthesis and/or increasing the concentration of cytotoxic kynurenine metabolites that act as agonists of the N-methyl-D-aspartate (NMDA)-glutamate receptors^19,28,29^. However, the relationship between dietary TLR variation and the kynurenine pathway activation has not been explored in detail.

We used UK Biobank resources (Application No: 1602) to determine dietary TLR based on food intake from the Oxford WEbQ online dietary questionnaire data and relate it to mood symptoms. Next we tested our hypothesis that those with a low TLR diet will be more vulnerable to risk genetic variants than those with a high TLR diet. To improve our genetic association results we combined several available genetic pathway resources to create a set of genes that fully represents serotonin and kynurenine pathway function. We further included regulatory variants with potential effects on the serotonin and kynurenine genes.

## Results

### Descriptive population statistics

Descriptive statistics are presented in Table 1 for low and high dietary TLR subgroups (N_TLR_Low_ = 36,038 and N_TLR_High_ = 27,239 respectively) and for the total population (N = 63,277). The distribution of age is similar in both low and high dietary TLR groups with a mean age of 57.0 and 56.8 respectively (t-test for difference of means, p-value: 0.009). However, there is a small difference between the two groups in the distribution of sex (low dietary TLR: 53.4% female, high dietary TLR: 55.6% female, which is trivially small but highly significant owing to the large sample size (chi-square p-value: 2.006e−08). The current depressive symptoms mean score is only marginally higher (t-test p-value: 0.037) in case of low dietary TLR (1.356) than that of high dietary TLR (1.348). In addition, the mean dietary TLR value in the low dietary TLR group is 0.041, whereas in case of the high dietary TLR group it is 0.070. In summary, the dietary TLR groups were closely matched in terms of age, sex and depressive symptoms but differences were statistically significant owing to the large sample sizes.

**Table 1:** Descriptive statistics for low and high dietary TLR subgroups and the total population.

Variables	Categories	All	Tryptophan / LNAA ratio (TLR) subgroups	
			Low dietary TLR	High dietary TLR
Population		63,277 (100%)	36,038 (57%)	27,239 (43%)
Sex	Female	34,377 (54.3%)	19,230 (53.4%)	15,147 (55.6%)
Age	Mean	56.93	56.99	56.83
	sd	7.84	7.79	7.89
Current depressive symptoms	Mean	1.352	1.356	1.348
	Sd	0.476	0.481	0.470
Tryptophan / LNAA ratio	mean	0.053	0.041	0.070
	sd	0.029	0.008	0.037

*LNAA* large neutral amino acids, *TLR* tryptophan to other LNAA ratio, *sd* standard deviation.

### Relationship between dietary TLR and depressive symptoms

The results of the linear regression model for current depressive symptoms score with age (beta = − 0.0094, se = 0.0002, p < 2E−16) and sex (beta = − 0.049, se = 0.0038, p < 2E−16) as covariates indicate that high dietary TLR has a moderate protective effect (beta = − 0.0106, se = 0.0038, p = 0.0049) with respect to depression. In addition, to support further analysis, a regression model with a continuous dietary TLR was also investigated with age (beta = − 0.0094, se = 0.0002, p < 2E−16) and sex (beta = − 0.049, se = 0.0038, p < 2E−16) as covariates in the model. Results confirmed the moderate protective effect of higher dietary TLR (beta = − 0.1644, se = 0.0641, p = 0.0103).

### SNP-heritability and genetic correlation of depressive symptoms and dietary TLR

In our study, depression has a similar SNP-heritability (h^2^ = 8.03%) as in a recent huge depression GWAS meta-analysis (h^2^ = 8.9%)^26^, whereas that of tryptophan intake (assessed through the continuous dietary TLR score) is smaller (h^2^ = 1.43%). Correcting for low/high dietary TLR in the models used for the analysis of the genetic background of depression had no apparent influence on the SNP-heritability of depression (h^2^ = 8.04%). This effect was also observed when dietary TLR ratio was added as a continuous covariate (h^2^ = 8.01%). Depression SNP-heritability was somewhat higher in the low dietary TLR group (h^2^ = 6.64%) compared to the high dietary TLR group (h^2^ = 4.1%). Genetic correlation between depression and the low/high dietary TLR was r_G_ = 0.1162 (SE = 0.1399, p > 0.05), indicating that there is no significant overlap between the genetic background of the two phenotypes. This relative genetic independence indicates that the dietary TLR effect on depression cannot be explained by overlapping genetic background.

### Genetic risk factors of depressive symptoms in low and high dietary TLR groups

#### SNP-level analysis

**Figure 1:**
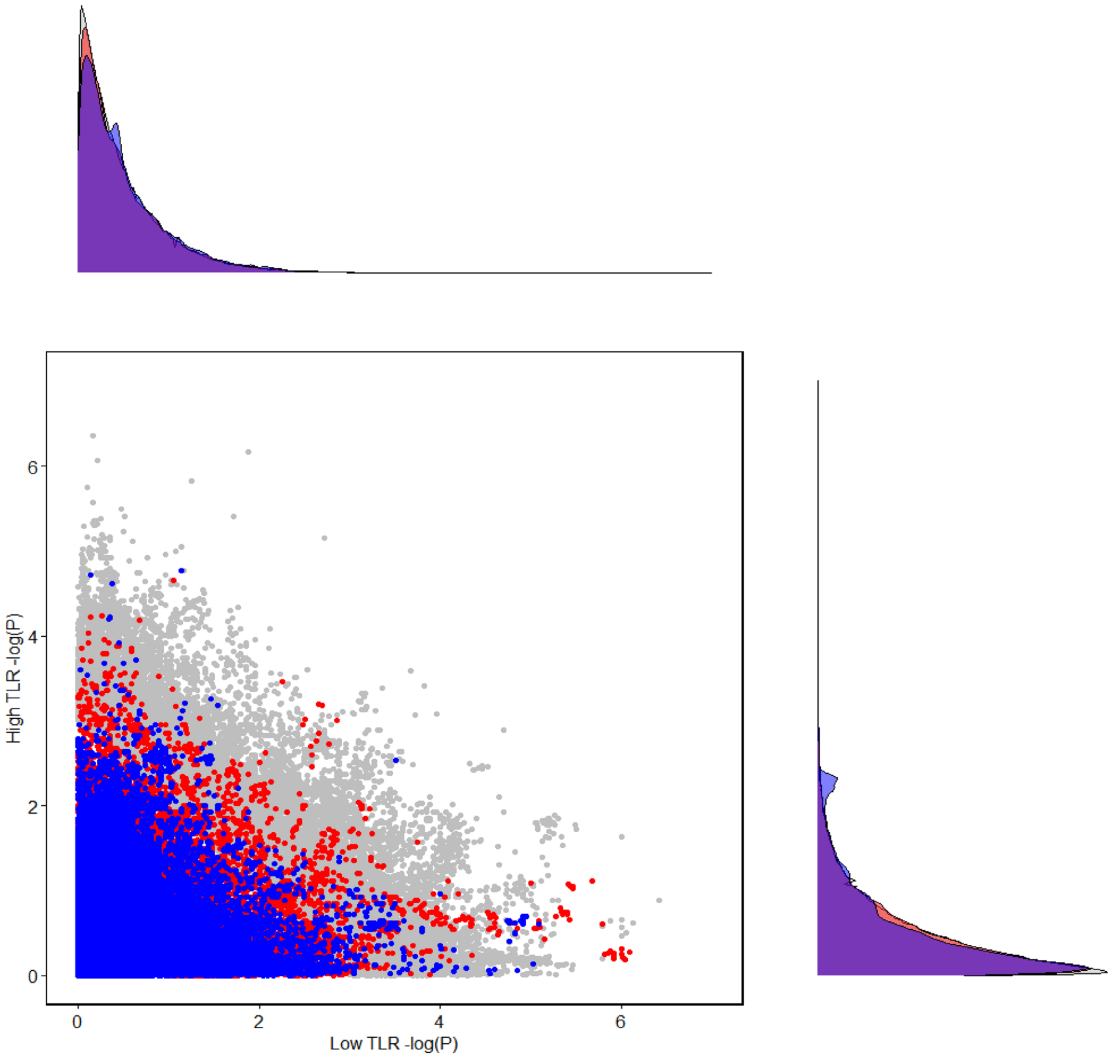
SNP association (p-value) scatterplot for depressive symptoms in low versus high dietary TLR subgroups showing the kynurenine-related SNPs in blue, the serotonin-related SNPs in red, and all the other SNPs in grey on a negative base ten logarithmic scale (− log(P)). TLR denotes dietary tryptophan/large neutral amino acids ratio. Density functions for SNP p-values are displayed on both axes, for kynurenine (blue) and serotonin (red) pathways. Note that the layer of blue dots obscures other dots in the lower left corner of the figure.

Regression analysis was performed using BOLT-LMM^30^ with respect to depressive symptoms in the total population, and in the dietary TLR subgroups. Results indicate that there are multiple SNPs related to the serotonin pathway that are associated with depressive symptoms with a suggestive significance (p < 3.36E−05) in the low dietary TLR subgroup, while no serotonin SNP reached this significance threshold in the high dietary TLR subgroup (see Fig. 1).

Regarding the kynurenine pathway, two SNPs were found to be associated with depressive symptoms with suggestive significance (p < 8.48E−05) in the low dietary TLR subgroup: rs4471056 (p = 8.10E−06, BOLT-LMM) and rs149007785 (p = 9.50E−06, BOLT-LMM). For additional details see Supplementary Information (Sect. 3.1), and Supplementary Tables S1, S2, and S3. Figure 1 shows that none of the serotonin- or kynurenine-related SNPs that were suggestively significant in the low dietary TLR subgroup showed significant or suggestive association with depressive symptoms in the high dietary TLR subgroup.

In the total population, two serotonin (rs2509453, p = 5.70E−06; rs2422859, p = 9.70E−06, BOLT-LMM) and one kynurenine (rs72740272, p = 3.50E−06, BOLT-LMM) pathway-related SNPs were found to be associated with depressive symptoms with suggestive significance. Note that these were not significant in either of the dietary TLR subgroups. See Supplementary Fig. S1a for the comparison of results related to the low dietary TLR subgroup versus those of the total population, and Supplementary Fig. S1b for the high dietary TLR subgroup versus the total population. In addition, GWAS SNP Manhattan plots for low and high dietary TLR subgroups and for total population are shown in Supplementary Fig. S1c–e respectively. For SNPs outside of our candidate pathways see Supplementary Information (Sect. 3.2).

#### Gene-level analysis

**Figure 2:**
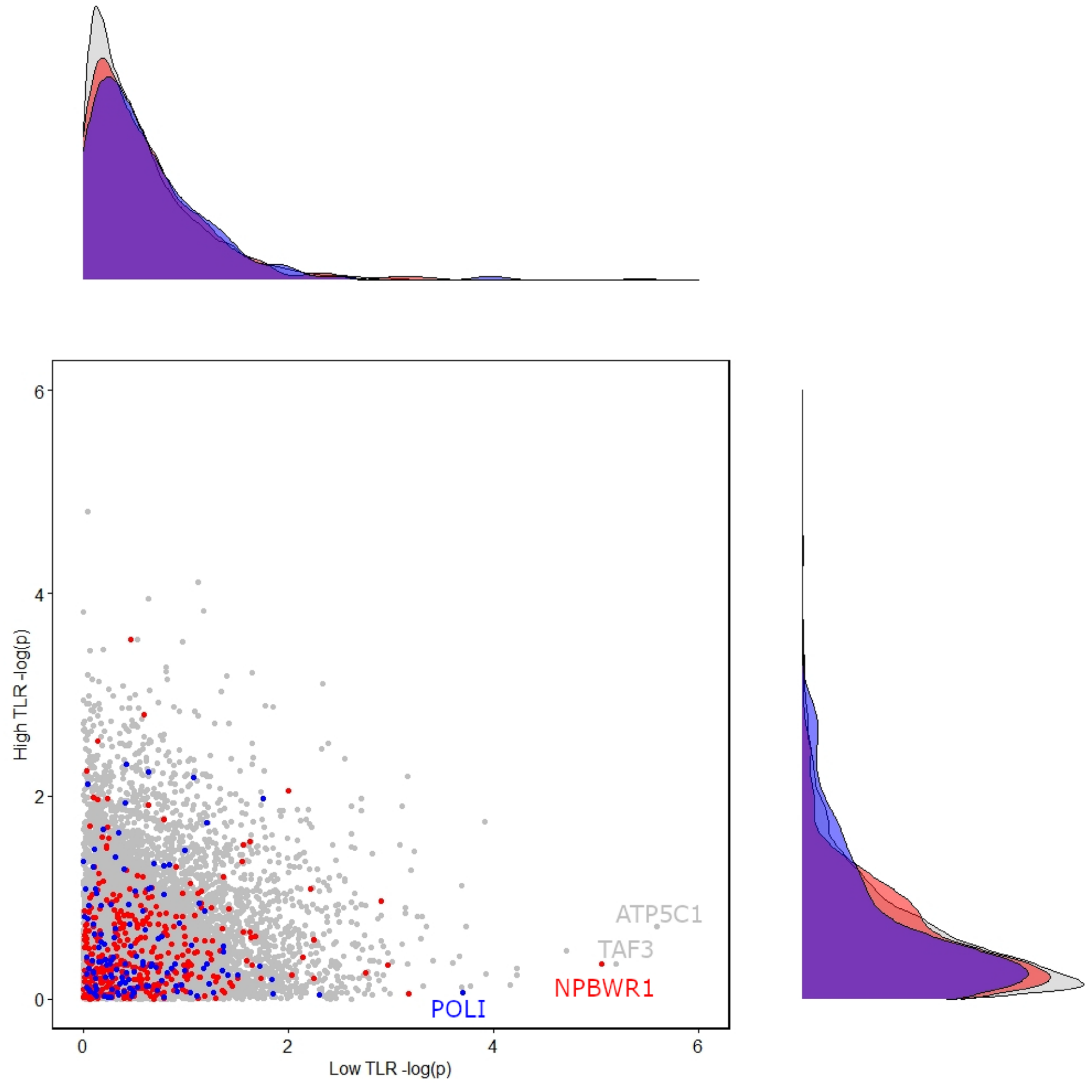
Gene-level association (p-value) scatterplot for depressive symptoms in low versus high dietary TLR subgroups showing kynurenine-related genes in blue, serotonin-related genes in red, and all the other genes in grey on a negative base ten logarithmic scale (− log(p)). *ATP5C1*, *NPBWR1* and *TAF3* genes survived gene-level correction for all genes in the low dietary TLR subgroup. In addition, *the POLI gene* survived correction for the kynurenine gene-set in the low dietary TLR subgroup.

Gene-level association p-values with respect to depressive symptoms were determined using the set-test method^31^. Results indicate that among the serotonin pathway genes only the *NPBWR1* (neuropeptides B and W receptor 1) is significantly associated at gene level with depressive symptoms in the low dietary TLR subgroup (p = 8.9E−06, set-test). Regarding the kynurenine pathway, the only gene significantly associated with depressive symptoms is *POLI* (DNA polymerase iota) again in the low dietary TLR subgroup (p = 2.0E−4, set-test). No other gene was significant after correcting for the set size, neither in the low nor in the high dietary TLR subgroup (see Supplementary Tables S4 and S5). In addition, Fig. 2 compares gene-level results between low and high dietary TLR subgroups. Further comparisons are shown in Supplementary Fig. S2a,b. For additional information concerning genes *NPBWR1* and *POLI* see Supplementary Information (Sect. 3.3).

Note that canonical serotonin related genes, such as serotonin receptor encoding *HTR1A* and *HTR2A*, serotonin transporter *SLC6A4*, and tryptophan hydroxylase encoding *TPH1* and *TPH2*, showed no significant association with depressive symptoms in any of the dietary TLR subgroups (see Supplementary Table S6). For further information concerning genes outside our candidate pathways see Supplementary Information (Sect. 3.4).

#### Pathway-level analysis

Pathway-level analysis was also performed with the set-test method based on the whole SNP set of the pathways (defined in the Methods section). Candidate pathways were tested for association with depressive symptoms. In the low dietary TLR subgroup and also in the whole population a significant association of serotonin (set-test p = 6.13E−06 and p = 4.92E−05 respectively) and kynurenine (set-test p = 0.0020 and p = 0.0093 respectively) main pathways with depressive symptoms were found, surviving candidate pathway-level correction (with a significance threshold of p-value < 0.017). In line with our hypothesis, however, no association was found in the high dietary TLR subgroup (see Table 2). Furthermore, to examine which part of the candidate pathways contributed the most to depressive symptoms we also tested candidate pathway components, i.e. subpathways (for details see Supplementary Information (Sect. 3.5) and Supplementary Table S7.

**Table 2:** Pathway- and subpathway-level set-test p-values for depressive symptoms for candidate pathways and their components with regard to dietary TLR subgroups.

	Low TLR (set-test p-value)	High TLR (set-test p-value)	Total population (set-test p-value)
Kynurenine	**0.002**	1.000	**0.009**
Kynurenine ChEMBL	0.038	1.000	1.000
Kynurenine GO	0.554	0.788	1.000
Kynurenine WP	0.101	0.544	0.054
Kynurenine Reactome	1.000	0.473	1.000
Serotonin	**6.13E−06**	1.000	**4.92E−05**
Serotonin GO	0.068	1.000	0.906
GO—serotonin receptor signalling pathway	0.029	1.000	1.000
GO—serotonin metabolic process	0.732	1.000	1.000
GO—serotonin binding	0.125	1.000	1.000
Reactome GPCR downstream	0.002#	1.000	0.074
Reactome serotonin	0.018	1.000	1.000
Serotonin WP	0.001#	1.000	1.000

Values in bold indicate pathway-level significance for the serotonin and kynurenine pathways (p < 0.017), (#) denotes subpathway-level significance for serotonin pathway components (p < 0.0021). TLR denotes tryptophan to other LNAA ratio.

In addition, we tested all biological process GO term-associated gene sets (with more than 10 genes), and we found that there were significant associations with depressive symptoms for GO terms such as “cerebral cortex cell migration”, and “adaptive thermogenesis” in the low dietary TLR subgroup (see Table 3 and Supplementary Tables S8 and S9).

**Table 3:** Pathway-level associations with depressive symptoms for the 20 most significant GO terms.

GO ID	Description	Low dietary TLR (set-test p-value)	High dietary TLR (set-test p-value)	Total (set-test p-value)
GO:1990845	Adaptive thermogenesis	**2.21E−06**	1.00E+00	7.16E−01
GO:0021795	Cerebral cortex cell migration	**2.76E−06**	1.43E−01	5.93E−04
GO:0043534	Blood vessel endothelial cell migration	**3.03E−06**	1.36E−03	**2.19E−07**
GO:0046683	Response to organophosphorus	**4.42E−06**	1.00E+00	**2.18E−06**
GO:0120162	Positive regulation of cold induced thermogenesis	**4.74E−06**	1.00E+00	1.60 E−03
GO:1901185	Negative regulation of erbb signaling pathway	**5.84E−06**	4.21E−01	1.16E−03
	Serotonin (candidate pathway)	**6.13E−06**	1.00E+00	4.92E−05
GO:2000177	Regulation of neural precursor cell proliferation	**6.40E−06**	8.42E−01	3.81E−01
GO:0001659	Temperature homeostasis	1.08E−05	1.00E+00	**1.30E−06**
GO:0007405	Neuroblast proliferation	1.08E−04	7.36E−02	**6.92E−06**
GO:0021543	Pallium development	1.21E−04	4.86E−03	**4.12E−09**
GO:0034446	Substrate adhesion dependent cell spreading	6.99E−04	8.73E−01	**5.08E−06**
GO:0030534	Adult behavior	1.23E−03	6.96E−02	**4.73E−09**
GO:0009409	Response to cold	2.44E−03	8.67E−02	**4.74E−06**
GO:1903169	Regulation of calcium ion transmembrane transport	5.39E−03	**4.37E−06**	2.00E−04
GO:0008344	Adult locomotory behavior	1.85E−02	2.56E−01	**1.65E−06**
GO:0043484	Regulation of rna splicing	3.30E−02	1.83E−04	**7.21E−06**
GO:0090092	Regulation of transmembrane receptor protein serine threonine kinase signaling pathway	3.45E−02	2.24E−05	**2.96E−07**
GO:0017038	Protein import	4.17E−02	8.32E−03	**2.51E−06**
GO:0033137	Negative regulation of peptidyl serine phosphorylation	9.93E−01	**3.24E−06**	5.53E−02
GO:0035567	Non canonical wnt signaling pathway	1.00E+00	3.25E−04	**2.88E−06**

Values represent set-test p-values with regression-based set size correction.

Values in bold indicate significant associations surviving pathway-level correction for GO terms (p < 7.39E−06).

**Figure 3:**
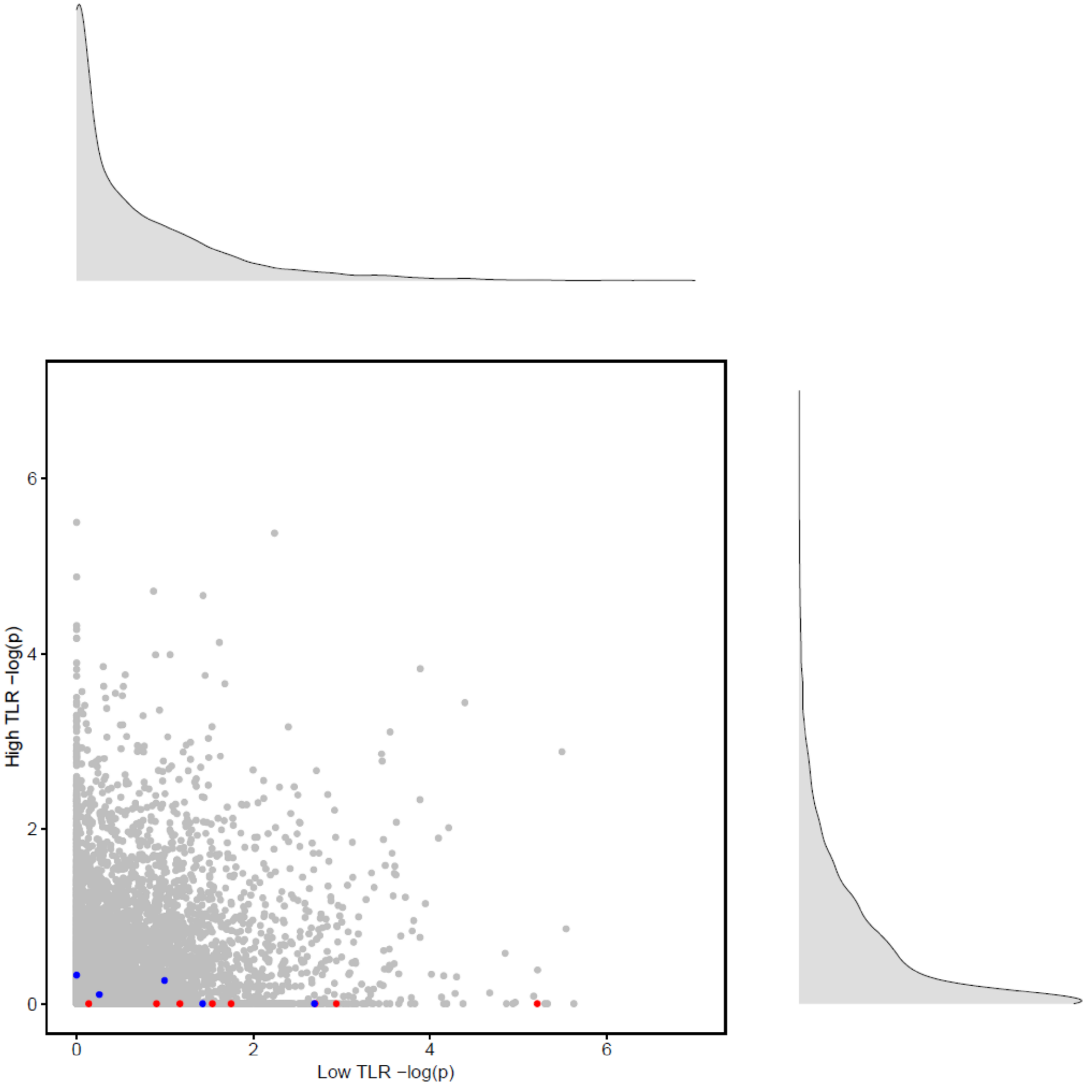
The pathway-level association (p-value) scatterplot for depressive symptoms in low versus high dietary TLR subgroups showing kynurenine-related pathways in blue, serotonin-related pathways in red, and p-values of biological process-related GO terms in grey on a negative base ten logarithmic scale (− log(p)).

Figure 3 shows for low and high dietary TLR subgroups the comparison of kynurenine and serotonin pathway-level associations with depressive symptoms and other GO terms’ results with set-size correction. For further comparisons see Supplementary Fig. S3a,b (with correction) and Supplementary Fig. S4a–c (without correction).

## Discussion

Our study is the first to investigate the effect of dietary TLR on genetic risk of depression using genome-wide genetic data. The main results corroborate our hypothesis that low dietary TLR reveals serotonin and kynurenine—related genetic risk of depressive symptoms which are not detectable in the high dietary TLR group. Furthermore, we identified multiple significant genetic pathways, including “adaptive thermogenesis”, “cerebral cortex cell migration”, “negative regulation of ERBB signalling”, “regulation of neural precursor cell proliferation”, in the background of depressive symptoms in the low dietary TLR subgroup, some of them being significant also in the total sample, while only two genetic pathways, “regulation of calcium ion transmembrane transport” and “negative regulation of peptidyl serine phosphorylation” showed significant associations in the high dietary TLR subgroup, with no overlap between the high dietary TLR subgroup and the total sample. Interestingly, the serotonin pathway was 7th in the rank (out of 6775 investigated GO terms and pathways) in the low dietary TLR subgroup and 30th in the total study population and the association between the serotonin pathway and depressive symptoms survived a correction at whole genome level (determined by FDR < 0.05) in the low dietary TLR subgroup. The success of stratification of the investigated population by a well-known metabolic pathway, here using dietary TLR, may be a useful approach to reveal genetic risk factors and biological mechanisms of depression.

Corroborating some former results^20,21^, we demonstrated an inverse relationship between habitual dietary TLR and depressive symptoms, although the effect size was small. This is also in line with previous experimental studies which demonstrated only modest effect in alteration of affective symptoms after dietary manipulations of TLR, i.e. increased affective symptoms after acute tryptophan depletion, and decreased symptoms following tryptophan augmentation e.g. via carbohydrate-rich diet^15,32,33^. There might be several explanations for this small or modest effect observed in previous studies, one of which is the complexity of diet that might interfere with mental health^34^. People with low dietary TLR tend to prefer a high protein and low carbohydrate diet, as was the case also in our study (see Supplementary Table S10). Detailed analysis of LNAA and tryptophan intake reveals that the intake of each LNAA amino acid (and also their sum) is significantly higher in the low dietary TLR subgroup than in the high dietary TLR subgroup (p < 2.20E−16, t-test), and similarly the tryptophan intake is also significantly higher (p = 5.06E−08, t-test) in the low dietary TLR subgroup, although to a lesser extent (see Supplementary Table S18). Even though tryptophan intake is slightly higher in the low dietary TLR subgroup, proportionally it is lower with respect to LNAA and thus the computed dietary TLR is lower. However, food intake resulting in low dietary TLR also comes with increased polyunsaturated fatty acid, vitamin D, vitamin B6 and vitamin B12 intake in our study that can counterbalance some of its effects. For example, polyunsaturated fatty acids^35^ and vitamin D^36^ might be able to diminish the effect of low TLR by decreasing inflammation. On the other hand, when considering C-reactive protein (CRP) level as an indicator of inflammation, the low dietary TLR subgroup has a significantly higher (p < 2.20E−16, t-test) plasma CRP than the high dietary TLR subgroup (see Supplementary Table S19), although it does not reach clinically relevant elevation but may suggest chronic low grade inflammation. Interestingly, none of the inflammation related GO terms had a significant association with depressive symptoms in neither of the dietary TLR subgroups. This may indicate that on a genetic level there is no direct relationship between current depressive symptoms and inflammation, but on a phenotypic level there is a possibility of a cross-talk between the effects of low dietary TLR and inflammation having an effect on current depressive symptoms. Although the exact mechanism is not well understood, other recent studies also suggest the important regulatory role of tryptophan metabolism in inflammatory processes that could be a fruitful drug target not only in depression and neuropsychiatric disorders but also in other inflammation related conditions^37,38^.

Another important explanation of the modest effect of dietary TLR on depressive symptoms could be supported by the observations based on depletion and supplementation studies. In those, the effect of changes in the acute intake of TLR modifying supplementation is not universal but highly dependent on vulnerability to depression^15,23,39^. Most sensitive people were either those who were healthy family members of depressed patients, or those who had more severe (chronic or recurrent) depressive episodes, or who were treated with selective serotonin reuptake inhibitors (SSRIs) pointing out the importance of genetic liability and perturbation of the serotonin system^33,40,41^. Thus future dietary prevention of mood disorders should be personalised based on genetic vulnerability and focused on complex dietary pattern instead of add-on nutrients.

Furthermore, we discovered that while several SNPs across the genome emerged as suggestively significant for depressive symptoms in low or high dietary TLR subgroups, serotonin or kynurenine SNPs showed such significance only in the low dietary TLR subgroup. Similarly, variants in a wide variety of serotonin and kynurenine pathway definitions were associated with depressive symptoms only in the low dietary TLR subgroup. We also presented evidence that this association is not due to a genetic correlation between dietary TLR and depressive symptoms. Sporadic and so far unreplicated studies have reported that variants in individual serotonin synthesis, transporter and receptor genes influence the response to tryptophan depletion. However, no previous studies assessed the role of a comprehensive set of serotonin pathway variants in interaction with habitual dietary TLR in a population cohort. Interestingly, although a shift in tryptophan metabolism towards the neurotoxic metabolites of the kynurenine pathway has long been implicated in the pathophysiology of depression^42–44^, there are no reports that kynurenine pathway genetic variants affect responses to experimental tryptophan manipulations.

Concerning the necessity of pathway-level approach, most large meta-analyses and genome-wide association studies of the genetics of major depressive disorder and depressed mood have not corroborated early reports of involvement of canonical serotonin genes for synthesis, reuptake and metabolism in depression^26,45^. Our results concur but at the same time clearly demonstrate significant involvement of the serotonin pathway, and a moderately significant role of the kynurenine pathway, in the development of depressive symptoms in those with low TLR and also in the total population. We show that perturbation in the serotonin and kynurenine systems can be detected at the pathway-level by taking into account downstream signalling mechanisms, regulatory and interacting biological processes, which are not comprehensively represented in the publicly available pathways but can be investigated by merging existing information.

Our results are in line with those studies which could not replicate candidate variant/gene findings in large cohort studies^46,47^.

Recently, in spite of the increased understanding that several systems and pathways beyond monoamines play a role in the development of depressive symptoms, multiple lines of evidence support the validity of a refined role for the serotonin system in depression. From serotonin tone during early life influencing development including the maturation and differentiation of brain pathways, through impacting sensitivity to stress, to adult emotion regulation, the serotonin system is implicated as a central player in depression^48^. Several lines of animal studies support that modification of any components of the serotonin system including its enzymes, receptors, downstream or other regulatory elements lead to depression-like behaviours or phenotypes^7^, and the effectiveness of serotonergic antidepressants, at least in a large subpopulation of depressive patients, likewise support the involvement of serotonin pathways in mood disorders^8^. Similarly, peripheral mechanisms involved in increased kynurenine production are becoming the focus of attention as kynurenine and its metabolites emerge as key neurobiological mediators in various physiological and pathological neuropsychological conditions and neuropsychiatric disorders including depression^34^, especially with the enhanced interest in the role of inflammatory processes which appear to affect mood via activation of the kynurenine pathway^44,49^. Rodent experiments show that blocking the activation of the kynurenine pathway reverses depression-like behaviour induced by various models. In humans, increased kynurenine pathway metabolites in the plasma and cerebrospinal fluid as well as increased kynurenine levels have shown an association with several measures of depression, including onset, symptom severity, anhedonia, loss of motivation, response to SSRIs or suicide attempt in different populations and studies^28,50^. Rodent studies suggest that central nervous system kynurenine concentrations are determined by peripheral kynurenine metabolism^28^, which among others influence saturation of the large neutral amino acid transporter, thus understanding the role of kynurenine in depression and potentially exploiting it for influencing risk and treatment would include understanding not only its actions in the brain but also in the periphery.

Beyond proving that depressive symptoms are associated with the serotonin and kynurenine pathway, especially in case of habitual food intake leading to low TLR, we have also demonstrated that when considering pathways reflected in biological process-related GO terms, the serotonin pathway shows a robustly strong significant association with depression only in the low dietary TLR subgroup, a much weaker association in the total sample and no association at all in the high dietary TLR subgroup. In addition, we have also identified several genome-level significant pathways in the low dietary TLR subgroup and to a lesser extent also in the total sample, which reflect divergent mechanisms associated with depression depending on dietary TLR.

For further details, see Supplementary Information (Sect. 3.6).

These results suggest that when placing serotonin and kynurenine in biological context, the effect of vulnerability genes on depression is more manifested in those with low but not with high dietary TLR. Therefore, based on previous studies^17–19,39^ and also supported by our results, the effect of food intake leading to low TLR on mood seems to be related to specific genetic risk mechanisms, including the serotonin and kynurenine pathways and mechanisms involved in adult neurogenesis, while higher tryptophan intake may have a broader, less specific effect on general health. Most importantly, our findings underline the importance of stratifying in analyses according to environmental and lifestyle factors such as dietary TLR level, also to identify distinct mechanisms in depression associated with potentially modifiable and influenceable lifestyle and nutritional factors.

However, this methodology of determining candidate pathway-level significance in subpopulations has multiple limitations. First of all, as all GWAS methods, it is sensitive to population stratification, i.e. the structure of population descriptors and phenotypes, therefore to some extent they are sensitive to exogenous environmental factors, and the capability of controlling for such effects is limited to a certain degree. Also, there is no guarantee that the SNPs have biologically relevant functions, and the methods to estimate the cumulative effect of SNPs using heritability estimates are known to have limitations, and may overestimate the true effect. The genetic relations between participants in GWAS-es are also problematic, though LMM methods can minimize the genetic effect of relations between people. However, environmental effects other than TLR can still affect the overall results. The used set-level technique is capable of combining the benefits of the LMM and the aggregation methods to get a good measure for the set-level significances. However, it is still affected by some of the common biases of aggregation techniques, namely the ambiguity of set definitions, and the difficulty of assigning effect size for significant results. Furthermore, in many cases, especially in case of mental illnesses, the phenotypes have significant biases. In addition, the derived TLR phenotype is computed using amino acid intake descriptors which are reasonable estimates based on food intake variables, however, it may contain biases due to the nature of its estimation. For example, we did not assess the timing of dietary intake and depressive symptoms, therefore we cannot make grounded conclusions on the direction of effects. However, the questionnaire we used has been validated in determining a habitual dietary pattern^51^. TLR could be determined more directly based on plasma tryptophan and plasma LNAA concentration levels, but such measurements were not available in our dataset. The utilized dietary TLR phenotype is an estimate based on a validated questionnaire. In addition, since there is no gold standard threshold for dietary TLR we applied a threshold based on our data. Furthermore, due to the nature of the dataset, whose main purpose was to facilitate genetic association analysis, several additional factors, e.g. urinary nitrogen excretion, were not taken into consideration, which could potentially provide further insight on results. Finally, despite that there are indications that the effect of dietary TLR is stronger in females we have not analysed males and females separately (sex was a covariate in all analyses) to increase sample size within TLR subgroups.

All in all, our study is the first to demonstrate that genetic variants and genes belonging to the serotonin and kynurenine pathways contribute to the genome-level risk of depression, but mainly in those who habitually follow a low TLR diet, and therefore have decreased tryptophan brain availability. Our comprehensive definition of serotonin and kynurenine gene sets enabled us to uncover that the revealed genes represent downstream signalling mechanisms and regulatory biological processes rather than classical candidate genes (such as transporters, receptors or enzymes), in line with recent extensive GWAS studies. However, our results also pointed out that processes related to neurodevelopment and adult neurogenesis, can be identified in a subgroup of subjects with such habitual food intake that leads to low TLR. Thus we think that our study provides the first genome-level link between the traditional serotonin hypothesis of depression and the recent GWAS results. From this point of view, our study design and results underpin the importance of selecting those moderator variables with well-known biological effect that really impact the emergence of genetic effects. Furthermore, by pointing out that lifestyle and dietary factors, such as dietary TLR, are directly related to the mechanisms associated with depression, our results, beyond highlighting the importance of considering such factors when focusing on the genetic background of depression, also emphasise the role of considering nutrition when approaching the treatment, and also possibly the prevention of depression from the direction of personalised medicine.

## Methods

### Study population

In this study, we investigated a subset of individuals from the UK Biobank resource (Application number: 1602), specifically those who completed both the enhanced touchscreen mental health questionnaire and the Oxford WEbQ dietary questionnaire. In addition, quality control steps for genetic measurements were performed. The final investigated subset consisted of 63,277 individuals, including 34,377 females (54.3%) and 28,900 males (45.7%) with a mean age of 56.93 (sd: 7.84) years. All procedures were carried out in accordance with the Declaration of Helsinki, and all individuals provided written informed consent. Ethical approval was given by National Research Ethics Service Committee North West–Haydock (11/NW/0382, 21/NW/0157).

### Derived variables

#### Depressive symptoms

The depressive symptoms score was determined based on current depressive symptoms available for subjects who completed the enhanced mental health questionnaire in the final two years of recruitment^52^. Measured symptoms consisted of frequency of (1) depressed mood, (2) unenthusiasm/disinterest, (3) tenseness/restlessness, (4) tiredness/lethargy in the last 2 weeks with respect to the survey. Each item was assessed on a scale of 1 (Not at all) to 4 (Nearly every day). A cumulative score of current depressive symptoms was computed as the sum of non-missing item scores divided by the number of scored items^53^.

#### Dietary tryptophan to other LNAA ratio (TLR)

Tryptophan and other LNAA intakes were estimated for subjects using the UK Biobank resource. Dietary nutrient descriptors were available for those who completed Oxford WEbQ, the online 24-h dietary recall questionnaire which assessed the estimated intake of various food types based on the consumed food and beverages excluding supplements^54–56^. Based on the available diet-related variables we estimated the tryptophan and other LNAA content of the diet of each subject by selecting the top 20 food sources^57^ from the National Diet and Nutrition Survey (https://www.gov.uk/government/statistics/ndns-results-from-years-5-and-6-combined), and then determined portion sizes using the Ministry of Agriculture, Fisheries and Food handbook^58^ (for details see Sect. 2.1 of Supplementary materials). The tryptophan/LNAA ratio (mean: 0.053 sd: 0.029) served as a measure of dietary habit. A cut-off value of 0.05 was selected based on the evaluation of our data and the consideration that there is no gold standard threshold for a “healthy” TLR according to previous tryptophan depletion and supplementation studies^32,59^. Based on this value two subgroups were created: an “unhealthy” group with relatively lower TLR (mean: 0.041, sd:0.008) which consisted of 36,028 individuals (57% of total), and a “healthy” group with relatively higher TLR (mean: 0.07, sd:0.037) which consisted of 27,239 individuals (43% of total).

### Genotyping and quality control methodology

To ensure the validity of results a quality control (QC) protocol was performed on genotypic data presented in Supplementary Fig. S5 following the UK biobank QC guidelines^60^. A detailed description of the protocol is provided in Sect. 2.2 of Supplementary materials.

### Gene and gene-set definitions

#### Gene definition

Gene position was determined using the ENSEMBL EMBL data in GRCh37 (https://www.ensembl.org/info/website/tutorials/grch37.html^61^). For each SNP in the imputed UK Biobank dataset we assigned all the corresponding genes where the gene-SNP relations were known. The position flag refers to the intron and exon regions of the genes between the start and end position defined by the EMBL. The regulatory annotation flags consist of the conventional 10 kilobase upstream and downstream regions as well as the three main databases with available variant-gene relations. These are (1) the GeneHancer V4_10^62^: containing transcription factor (TF), enhancer and silencer information, (2) GTEx_Analysis_v7_eQTL (https://www.gtexportal.org/home/): containing expression quantitative trait loci (eQTL) data, and (3) rSNP base v3.1 (http://rsnp.psych.ac.cn/^63^ containing various regulatory functions such as TF binding regions, chromatin interactive regions, topologically associated domains, long non-coding RNAs (lncRNAs) coding regions, mature microRNAs (miRNAs) regions, target sites of miRNAs, and circular RNA (circRNA) regions. Functional annotation of investigated serotonin and kynurenine SNPs is shown in Supplementary Tables S11 and S12.

#### Gene set definitions

Our aim was to investigate a set of genes that may link tryptophan intake with depression through serotonin signalling. As a first step, genes of canonical receptors and transporters, as well as genes of downstream signalling were examined from serotonin signalling pathways originating from Wikipathways (www.wikipathways.org^64^, Reactome Pathway Database (https://reactome.org/^65,66^, and GeneOntology (http://geneontology.org/^67,68^. Subsequent analysis revealed that there are considerable differences between the serotonin-related genes of these sources (for overlaps see Supplementary Fig. S6a), and that a unified approach is necessary. Therefore, based on these sources we defined a set of 391 genes (resources were accessed in March 2019, see Supplementary Table S13), which consists of the union of genes from different sources. Wikipathway (WP) entries included WP4017, WP3944, WP3947, WP732, WP722, WP734. The Reactome contained the downstream GPCR pathway and the serotonin pathways. From GeneOntology (GO) the following terms were used: GO:0007210 GO:0,099,589, GO:0042428, GO:1904014, GO:0006837, GO:0002351, GO:0051378, GO:0031821, GO:0099154.

Another way to link tryptophan intake to depression is via the kynurenine pathways consisting of canonical enzymes. The kynurenine enzymes originated from the Wikipathway WP4210 and WP465 pathways, from GeneOntology terms GO:0070189, GO:0006568, GO:0034275, GO:0030429, and from the tryptophan catabolism pathway described in Reactome. Due to the biological activity of the metabolites of this pathway, namely tryptophan, kynurenine, transtorine or kynurenic acid, quinolinic acid, and 2-picolinic acid, the non-specific binders of these metabolites, i.e. related enzymes, transporters, and receptors were also included in our set based on the ChEMBL Database (https://www.ebi.ac.uk/chembl/, CHEMBL25 March 2019, https://doi.org/10.6019/CHEMBL.database.25). Protein-metabolite binding represented by pchembl values (derived from K_a_, K_d_, IC50, K_i_ measures or their logarithmic values) were considered effective if the binding constants exceeded 10-times the concentration of metabolites normally present in the human brain^69–71^. The constructed kynurenine gene set consisted of 131 genes with ENSEMBL IDs (Resources were accessed in March 2019, see Supplementary Table S14, for overlaps in different sources see Supplementary Fig. S6b). Note that tryptophan metabolic processes branch into two fundamentally different paths; the serotonin pathway is a signalling pathway, while the kynurenine pathway is an enzymatic process. Therefore, downstream genes are present only in the serotonin set, while non-specific binders are present only in the kynurenine set. Furthermore, note that 9 genes were present in both the serotonin and kynurenine sets.

### Statistical analysis

#### Basic statistical methods

Descriptive statistics and regression models were computed using basic packages of the R statistical software (https://www.r-project.org/). Regarding genetic associations, significance thresholds were corrected for multiple testing based on the number of investigated SNPs, genes, or tested pathways. SNP- and gene-level thresholds were determined for serotonin and kynurenine pathway hypotheses separately (see Table 4).

**Table 4:** Corrected significance thresholds used in the study.

	All genetic data	Serotonin	Kynurenine
SNP			
Significance	5.00E−08	1.68E−07	4.24E−07
Suggestive	1.00E−05	3.36E−05	8.48E−05
Gene			
Significance	3.71E−06	1.28E−04	3.85E−04
FDR 0.1	1.49E−04	2.56E−04	7.6E−04

	GO terms	Serotonin	Kynurenine
Pathway			
Significance	7.39E−06	1.70E−02	1.70E−02
Candidate pathway components			
Significance	–	2.08E−03	3.33E−03

FDR denotes false discovery rate. *Serotonin* and *Kynurenine* pathways refer to our main candidate pathways. *GO terms* refer to Gene Ontology terms. Candidate pathway components refer to the corresponding candidate subpathways. *Serotonin* pathway components: Serotonin GO, GO—serotonin receptor signalling pathway, GO—serotonin metabolic process, GO—serotonin binding, Reactome GPCR downstream, Reactome serotonergic, Serotonin WP; *Kynurenine* pathway components: Kynurenine ChEMBL, Kynurenine GO, Kynurenine WP, Kynurenine Reactome.

The applied significance thresholds for SNPs are widely accepted in the literature, a corresponding suggestive significance threshold was computed by multiplying the strict threshold by 200^72^. Furthermore, alongside the restrictive significance levels, suggestive significance thresholds were determined for genes corresponding to a false discovery ratio (FDR) of less than 0.1, which means that less than 10% of these suggestive results are false positive. Note that due to the nature of the Benjamini–Hochberg method the p-value threshold corresponding to an FDR value is an approximation, because the exact threshold depends on the p-value distribution.

#### BOLT—LMM method

BOLT-LMM, a linear mixed model, was applied to perform association testing between depression and genotypes. This method uses a Bayesian mixture-of-normals prior to handle the random effect of SNPs (other than the SNP which is tested). The model is a generalized form of the standard “infinitesimal” mixed model with increased power. BOLT-LMM can compute mixed model association statistics faster than conventional methods, and provides a more accurate distribution for the resulting SNP-level p-values^30^.

#### Set-test method

For gene and pathway calculations a linkage disequilibrium (LD) pruning was performed on the variants to reduce the computational costs with no significant information loss. As a first step, SNPs without annotated genes were filtered out. Subsequently, an LD pruning was performed on the remaining SNPs with LD > 0.8 over 1500 bases with a 150-base long window. The resulting set of SNPs served as the ‘tested SNP sets’ for the *set-test method* which is an implementation of the set-based variance component tests^31^ method, aimed at testing the association between a set of SNPs and the target phenotype. It is based on a linear mixed model similar to the previously presented LMM technique, in this case, however, genetic background (relatedness), the joint effect of the SNP set, and residual noise are modelled as random effects with separate variance components. Estimating variance components amounts to estimating the variance explained by the SNP set (e.g. a gene of the candidate pathway), genetic background (e.g. polygenic effects, population structure or cryptic relatedness), and noise (e.g. environmental effects). Testing for association between the set and the phenotype can be performed by determining whether the corresponding variance component is significantly different from zero. In order to compute the ‘genetic background variance component’ of the model used by the set-test method, a ‘genetic background’ SNP set was determined based on their relevance with respect to depressive symptoms in the total population. First, an association test was performed on all SNPs with respect to the depressive symptoms phenotype, then based on that result an LD-based clumping was performed using the following parameters: 0.05 significance threshold, 0.1 LD threshold and 160 megabase physical distance. The resulting SNPs were used as the genetic background set.

#### Aggregations for gene and pathway levels

Set-test was used to directly calculate the gene-level p-values for each gene using the default LD pruning cut-off of 0.8. SNPs were aggregated to gene level in order to investigate their aggregate effect with respect to depressive symptoms. Note that such an aggregation leads to more robust results in general^73^. In cases when pathways were investigated, a regression-based correction was used to eliminate the effect of set sizes on the p-values (see Supplementary Table S7). Due to the stochastic nature of set-test computations, the resulting p-values were averaged over 10 runs in case of the genes and 5 runs in case of the pathways. Note that the calculated pathway-level p-values are presented in the results section only for sets with more than 10 genes due to the otherwise high overlap of pathways caused by the hierarchical nature of the GO terms.

### Heritability calculation

Heritability was computed using the LDSC software^74^ in two steps following the user manual. First, LD score was calculated for all genotyped SNPs using the post quality control data extended with genetic linkage information from 1000 Genomes Project (https://mathgen.stats.ox.ac.uk/impute/1000GP_Phase3.html). Second, h^2^ values were calculated in the LDSC software using the estimated LD scores and p-values computed by BOLT-LMM.

## Supplementary Information

Supplementary Information 1.

Supplementary Information 2.

Supplementary Information 3.

Supplementary Information 4.

## Data Availability

The datasets analysed in this study are available in the UK Biobank repository (https://bbams.ndph.ox.ac.uk/ams/), where registered researchers can apply for the use of the data. Generated summary statistics are available as attached supplementary data files (Supplementary Tables S15–S17).
