# Supplementary material for: Genetic risk of depression is different in subgroups of dietary ratio of tryptophan to large neutral amino acids
Source: Sci Rep. 2023 Mar 27;13:4976. doi: 10.1038/s41598-023-31495-x (PMC10042855; doi:10.1038/s41598-023-31495-x)
Supplement: Supplementary file 2 — Supplementary Information 2. [file 41598_2023_31495_MOESM2_ESM.zip › Supplementary File F2.pdf]

Supplementary File F2

Genetic risk of depression is different in subgroups of dietary ratio of tryptophan to large neutral amino acids

Bence Bruncsics[1,ǂ], Gabor Hullam[1,2,ǂ], Bence Bolgar[1,ǂ], Peter Petschner[2,3,4], Andras Millinghoffer[1,3], Kinga Gecse[2,5], Nora Eszlari[2,3], Xenia Gonda[3,6], Debra J Jones[7], Sorrel T Burden[7], Peter Antal[1], Bill Deakin[8], Gyorgy Bagdy[2,3], Gabriella Juhasz[2,3,5,*]

[1] *Department of Measurement and Information Systems, Budapest University of Technology and Economics, Muegyetem rkp. 3., H-1111 Budapest, Hungary*

[2] *Department of Pharmacodynamics, Faculty of Pharmacy, Semmelweis University, Budapest, Hungary*

[3] *NAP3.0-SE Neuropsychopharmacology Research Group, Hungarian Brain Research Program, Semmelweis University, Budapest, Hungary*

[4] *Bioinformatics Center, Institute for Chemical Research, Kyoto University, Gokasho, Uji, Kyoto, Japan*

[5] *SE-NAP2 Genetic Brain Imaging Migraine Research Group, Hungarian Brain Research Program, Semmelweis University, Budapest, Hungary*

[6] *Department of Psychiatry and Psychotherapy, Faculty of Medicine, Semmelweis University, Budapest, Hungary*

[7] *School of Health Sciences, University of Manchester, Manchester, UK*

[8] *Division of Neuroscience and Experimental Psychology, School of Biological Sciences, Faculty of Biology, Medicine and Health, University of Manchester, Manchester Academic Health Science Centre, Manchester, United Kingdom*

[ǂ] The authors contributed equally to the work.

[*] **Corresponding author:** Gabriella Juhasz

Postal address: Department of Pharmacodynamics, Faculty of Pharmacy, Semmelweis University, Budapest, Hungary, 1089 Budapest, Nagyvarad ter 4. Hungary.
Phone: +36-1-4591500/56362, Fax: +36-1-4591494

Due to their large size, **Supplementary Tables S11-S12** are provided as compressed files.

Supplementary Table S11

Serotonin SNPs (gene-SNP relations) with functional annotation based on position, GeneHancer, eQTL, and rSNPbase annotation.

Supplementary Table S12

Kynurenine pathway SNPs (gene-SNP relations) with functional annotation based on position, GeneHancer, eQTL, and rSNPbase annotation.

Supplementary Table Extra

All investigated genes and SNPs with annotation.

Annotation codes:

Pos - Positional information based on EMBL gene start and end positions

K10 - Conventional +- 10000 base before the gene start position and after the end position

rSNP - Gene SNP regulatory association based on rSNP base database

eQTL - SNP-gene eQTL annotation based on the GTEx

GH - Regulatory information based on the GeneHancer database

The first column contains the gene ENSEMBL ID followed by the SNP RS IDs associated to that gene with the annotations separated by a „|" sign.

Example:

ENSG000001 rs00001|Pos rs00002|Pos rs00001|GH
