# Supplementary material for: Genetic risk of depression is different in subgroups of dietary ratio of tryptophan to large neutral amino acids
Source: Sci Rep. 2023 Mar 27;13:4976. doi: 10.1038/s41598-023-31495-x (PMC10042855; doi:10.1038/s41598-023-31495-x)
Supplement: Supplementary file 3 — Supplementary Information 3. [file 41598_2023_31495_MOESM3_ESM.zip › Supplementary File F3.pdf]

Supplementary File F3

Genetic risk of depression is different in subgroups of dietary ratio of tryptophan to large neutral amino acids

Bence Bruncsics[1,ǂ], Gabor Hullam[1,2,ǂ], Bence Bolgar[1,ǂ], Peter Petschner[2,3,4], Andras Millinghoffer[1,3], Kinga Gecse[2,5], Nora Eszlari[2,3], Xenia Gonda[3,6], Debra J Jones[7], Sorrel T Burden[7], Peter Antal[1], Bill Deakin[8], Gyorgy Bagdy[2,3], Gabriella Juhasz[2,3,5,*]

[1] *Department of Measurement and Information Systems, Budapest University of Technology and Economics, Muegyetem rkp. 3., H-1111 Budapest, Hungary*

[2] *Department of Pharmacodynamics, Faculty of Pharmacy, Semmelweis University, Budapest, Hungary*

[3] *NAP3.0-SE Neuropsychopharmacology Research Group, Hungarian Brain Research Program, Semmelweis University, Budapest, Hungary*

[4] *Bioinformatics Center, Institute for Chemical Research, Kyoto University, Gokasho, Uji, Kyoto, Japan*

[5] *SE-NAP2 Genetic Brain Imaging Migraine Research Group, Hungarian Brain Research Program, Semmelweis University, Budapest, Hungary*

[6] *Department of Psychiatry and Psychotherapy, Faculty of Medicine, Semmelweis University, Budapest, Hungary*

[7] *School of Health Sciences, University of Manchester, Manchester, UK*

[8] *Division of Neuroscience and Experimental Psychology, School of Biological Sciences, Faculty of Biology, Medicine and Health, University of Manchester, Manchester Academic Health Science Centre, Manchester, United Kingdom*

[ǂ] The authors contributed equally to the work.

[*] **Corresponding author:** Gabriella Juhasz

Postal address: Department of Pharmacodynamics, Faculty of Pharmacy, Semmelweis University, Budapest, Hungary, 1089 Budapest, Nagyvarad ter 4. Hungary.

Phone: +36-1-4591500/56362, Fax: +36-1-4591494

Due to their large size, Supplementary Tables S15-S17 are provided as separate compressed files.

- **Supplementary Table S15**. SNP level association results with respect to depressive symptoms concerning the serotonin pathway for low and high TLR subgroups, and for the total population. P-values are displayed for low and high TLR subgroups and also for the total population.

- **Supplementary Table S16**. SNP level association results with respect to depressive symptoms concerning the kynurenine pathway for low and high TLR subgroups, and for the total population. P-values are displayed for low and high TLR subgroups and also for the total population.

- **Supplementary Table S17**. SNP level association results with respect to depressive symptoms concerning all SNPs for the total population, and for low and high TLR subgroups. P-values are displayed for low and high TLR subgroups and also for the total population.
