# Supplementary material for: Genetic risk of depression is different in subgroups of dietary ratio of tryptophan to large neutral amino acids
Source: Sci Rep. 2023 Mar 27;13:4976. doi: 10.1038/s41598-023-31495-x (PMC10042855; doi:10.1038/s41598-023-31495-x)
Supplement: Supplementary file 4 — Supplementary Information 4. [file 41598_2023_31495_MOESM4_ESM.pdf]

Genetic risk of depression is different in subgroups of dietary ratio of tryptophan to large neutral amino acids

Bence Bruncsics[1,ǂ], Gabor Hullam[1,2,ǂ], Bence Bolgar[1,ǂ], Peter Petschner[2,3,4], Andras Millinghoffer[1,3], Kinga Gecse[2,5], Nora Eszlari[2,3], Xenia Gonda[3,6], Debra J Jones[7], Sorrel T Burden[7], Peter Antal[1], Bill Deakin[8], Gyorgy Bagdy[2,3], Gabriella Juhasz[2,3,5,*]

[1] *Department of Measurement and Information Systems, Budapest University of Technology and Economics, Muegyetem rkp. 3., H-1111 Budapest, Hungary*

[2] *Department of Pharmacodynamics, Faculty of Pharmacy, Semmelweis University, Budapest, Hungary*

[3] *NAP3.0-SE Neuropsychopharmacology Research Group, Hungarian Brain Research Program, Semmelweis University, Budapest, Hungary*

[4] *Bioinformatics Center, Institute for Chemical Research, Kyoto University, Gokasho, Uji, Kyoto, Japan*

[5] *SE-NAP2 Genetic Brain Imaging Migraine Research Group, Hungarian Brain Research Program, Semmelweis University, Budapest, Hungary*

[6] *Department of Psychiatry and Psychotherapy, Faculty of Medicine, Semmelweis University, Budapest, Hungary*

[7] *School of Health Sciences, University of Manchester, Manchester, UK*

[8] *Division of Neuroscience and Experimental Psychology, School of Biological Sciences, Faculty of Biology, Medicine and Health, University of Manchester, Manchester Academic Health Science Centre, Manchester, United Kingdom*

[ǂ] The authors contributed equally to the work.

[*] **Corresponding author:** Gabriella Juhasz

Postal address: Department of Pharmacodynamics, Faculty of Pharmacy, Semmelweis University, Budapest, Hungary, 1089 Budapest, Nagyvarad ter 4. Hungary.

Phone: +36-1-4591500/56362

SUPPLEMENTARY INFORMATION

This supplementary information text file provides a list of all supplementary tables and figures (section 1), followed by additional descriptions related to methods applied in the study (section 2), and describes additional results (section 3).

1. List of supplementary tables and figures

1.1. Tables

- **Supplementary Table S1.** Suggestively significant SNPs with respect to depressive symptoms for the low TLR subgroup. The table displays the corresponding genes for each SNP and indicates whether a SNP is related to the serotonin or kynurenine pathways.
  This is provided as a part of the **Supplementary excel file F1**.

- **Supplementary Table S2**. Suggestively significant SNPs with respect to depressive symptoms for the high TLR subgroup. The table displays the corresponding genes for each SNP and indicates whether a SNP is related to the serotonin or kynurenine pathways.
  This is provided as a part of the **Supplementary excel file F1**.

- **Supplementary Table S3**. Suggestively significant SNPs with respect to depressive symptoms for the total population. The table displays the corresponding genes for each SNP and indicates whether a SNP is related to the serotonin or kynurenine pathways.
  This is provided as a part of the **Supplementary excel file F1**.

- **Supplementary Table S4**. Gene level association results with respect to depressive symptoms concerning the serotonin pathway for low and high TLR subgroups, and for the

total population. P-values and corresponding q-values are displayed for low and high TLR subgroups and also for the total population.
This is provided as a part of the **Supplementary excel file F1**.

- **Supplementary Table S5**. Gene level association results with respect to depressive symptoms concerning the kynurenine pathway for low and high TLR subgroups, and for the total population. P-values and corresponding q-values are displayed for low and high TLR subgroups and also for the total population.
  This is provided as a part of the **Supplementary excel file F1**.

- **Supplementary Table S6.** Gene level association results with respect to depressive symptoms concerning all genes for low and high TLR subgroups, and for the total population. P-values and corresponding q-values are displayed for low and high TLR subgroups and also for the total population.
  This is provided as a part of the **Supplementary excel file F1**.

- **Supplementary Table S7**. Candidate pathway and subpathway level associations for the serotonin and kynurenine pathways in case of low and high TLR subgroups, and for the total population. P-values and corresponding q-values are displayed without correction, and with correction for set size using a regression based method.
  This is provided as a part of the **Supplementary excel file F1**.

- **Supplementary Table S8.** Pathway level associations with respect to depressive symptoms for candidate pathways and GO terms with FDR<0.1 in the low TLR subgroup and/or in the total population. The table displays p-values with and without correction for set size using a regression based method, and corresponding q-values for low TLR and high TLR subgroups and for the total population.
  This is provided as a part of the **Supplementary excel file F1**.

- **Supplementary Table S9.** Pathway level associations with respect to depressive symptoms for candidate pathways and GO terms for low and high TLR subgroups, and for

the total population. The table displays p-values with and without correction for set size using a regression based method, and corresponding q-values for low TLR and high TLR subgroups and for the total population.

This is provided as a part of the **Supplementary excel file F1**.

- **Supplementary Table S10.** Nutrient intake in low and high TLR groups. Unit denotes the measurement unit for each variable as available in the UKB data. Difference of means (absolute) and Difference of means (relative vs low TLR) denote the absolute and relative difference between means of low and high TLR subgroups. Relative differences are shown with respect to the means of low TLR subgroup. Difference of means (p-value) displays the p-value related to the t-test for difference of means between low and high TLR groups for each variable. Sig. marks significant p-values taking into consideration a correction for multiple testing with a threshold of 0.0025. Markers (*), (**), and (***) denote statistically significant difference, highly significant difference (p< 1.0e-6), and clear difference (p< 2.2e-16) respectively.

- **Supplementary Table S11.** Serotonin SNPs (gene-SNP relations) with functional annotation based on position, GeneHancer, eQTL, and rSNPbase annotation.

  This is provided as **Supplementary text file F2 (compressed)**.

- **Supplementary Table S12.** Kynurenine pathway SNPs (gene-SNP relations) with functional annotation based on position, GeneHancer, eQTL, and rSNPbase annotation.

  This is provided as **Supplementary text file F2 (compressed)**.

- **Supplementary Table S13.** Selected genes for the serotonin pathway. The table lists the ENSEMBLE ID, official name, and the database source of selected genes.

  This is provided as a part of the **Supplementary excel file F1**.

- **Supplementary Table S14.** Selected genes for kynurenine pathway. The table lists the ENSEMBLE ID, official name, and the database source of selected genes.

  This is provided as a part of the **Supplementary excel file F1**.

- **Supplementary Table S15**. SNP level association results with respect to depressive symptoms concerning the serotonin pathway for low and high TLR subgroups, and for the total population. P-values are displayed for low and high TLR subgroups and also for the total population.
  This is provided as a part of the **Supplementary text file F3 (compressed)**.

- **Supplementary Table S16**. SNP level association results with respect to depressive symptoms concerning the kynurenine pathway for low and high TLR subgroups, and for the total population. P-values are displayed for low and high TLR subgroups and also for the total population.
  This is provided as a part of the **Supplementary text file F3 (compressed)**.

- **Supplementary Table S17**. SNP level association results with respect to depressive symptoms concerning all SNPs for the total population, and for low and high TLR subgroups. P-values are displayed for low and high TLR subgroups and also for the total population.
  This is provided as a **Supplementary text file F3 (compressed)**.

- **Supplementary Table S18.** LNAA and tryptophan intake in low and high TLR groups. Unit denotes the measurement unit for each variable as available in the UKB data. Difference of means (absolute) and Difference of means (relative vs low TLR) denote the absolute and relative difference between means of low and high TLR subgroups. Relative differences are shown with respect to the means of low TLR subgroup. Difference of means (p-value) displays the p-value related to the t-test for difference of means between low and high TLR groups for each variable. Sig. marks significant p-values taking into consideration a correction for multiple testing with a threshold of 0.0025. Markers (*), (**), and (***) denote statistically significant difference, highly significant difference ($p< 1.0e-6$), and clear difference ($p< 2.2e-16$) respectively.

- **Supplementary Table S19.** Plasma CRP level in low and high TLR groups. Difference of means (absolute) and Difference of means (relative vs low TLR) denote the absolute and relative difference between means of low and high TLR subgroups. Relative difference is shown with respect to the mean of low TLR subgroup. Difference of means (p-value) displays the p-value related to the t-test for difference of means between low and high TLR groups.

1.2. Figures

- **Supplementary Figure S1a.** Comparison of results related to the BOLT-LMM based association test of SNPs with respect to depression. The scatter plot displays -log10 p-values for the low TLR subgroup versus total population. Kynurenine and serotonin pathway related SNPs are denoted with blue and red markers respectively, other SNPs are denoted with grey markers.

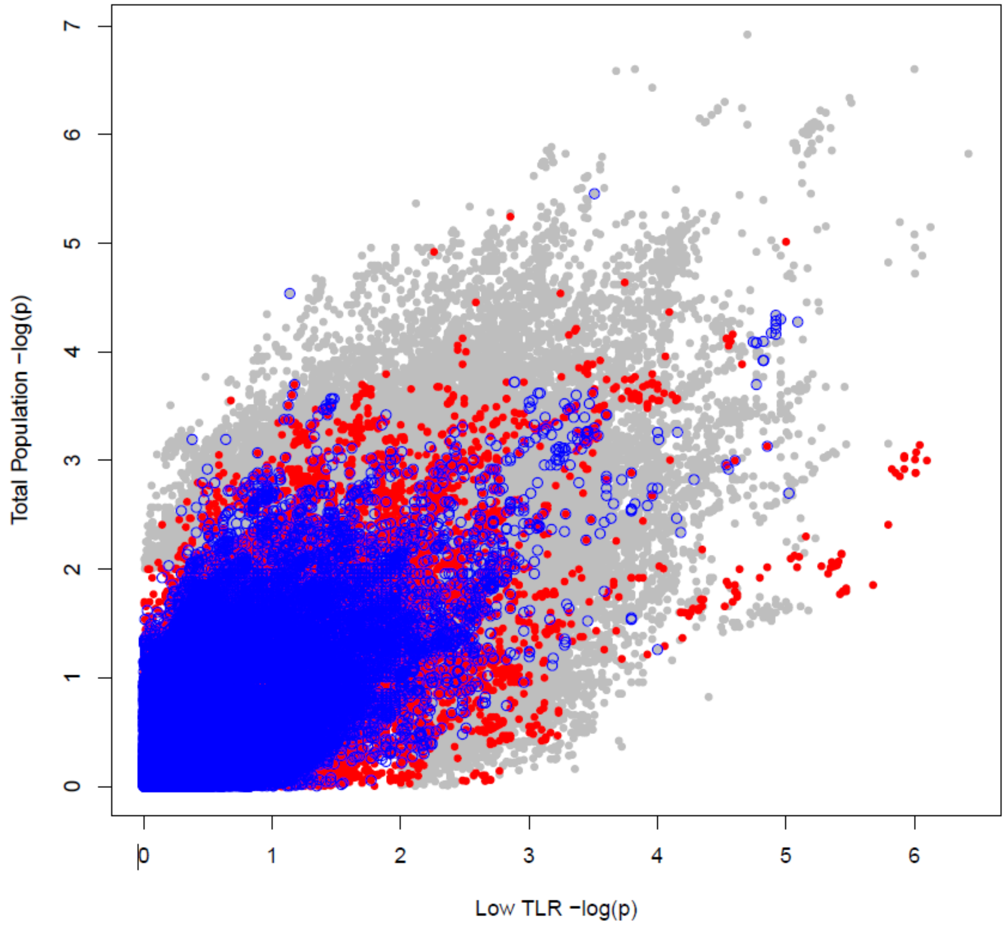

- **Supplementary Figure S1b**. Comparison of results related to the BOLT-LMM based association test of SNPs with respect to depression. The scatter plot displays -log10 p-values for the high TLR subgroup versus total population. Kynurenine and serotonin pathway related SNPs are denoted with blue and red markers respectively, other SNPs are denoted with grey markers.

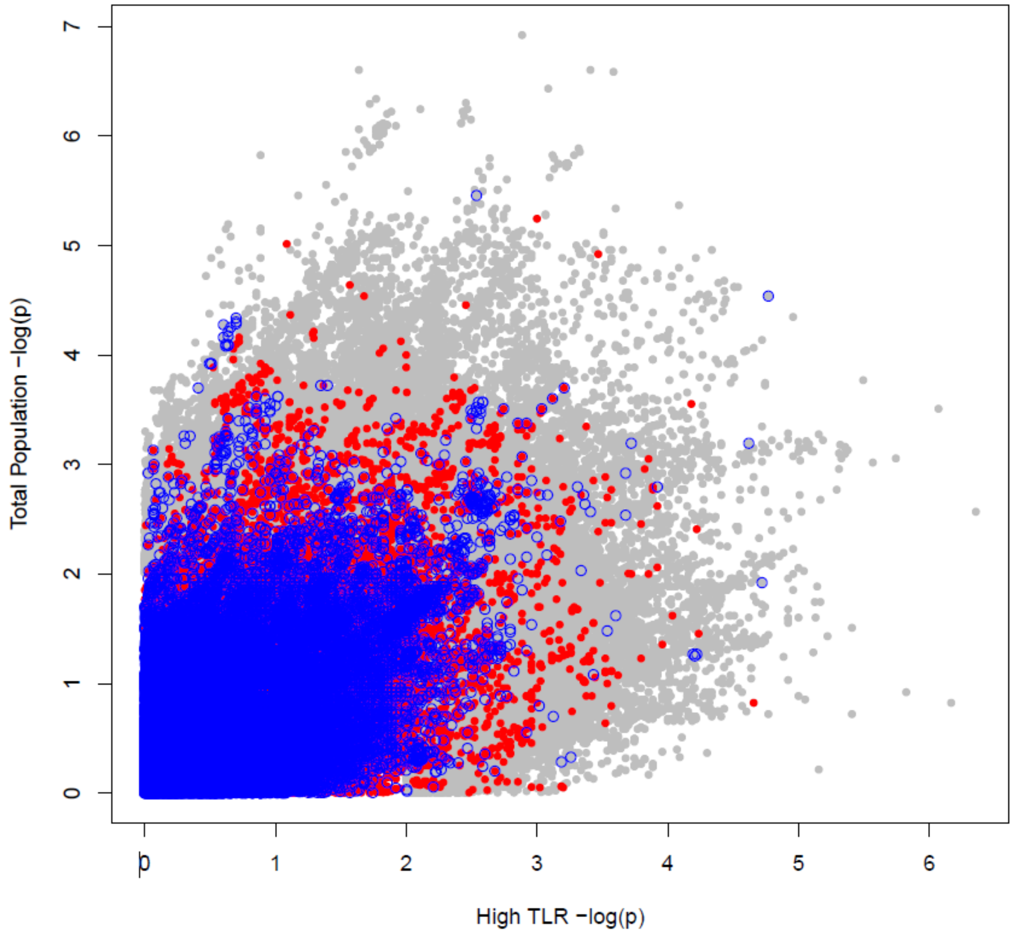

- **Supplementary Figure S1c**. SNP level Manhattan plots for GWAS analysis - low TLR subgroup.

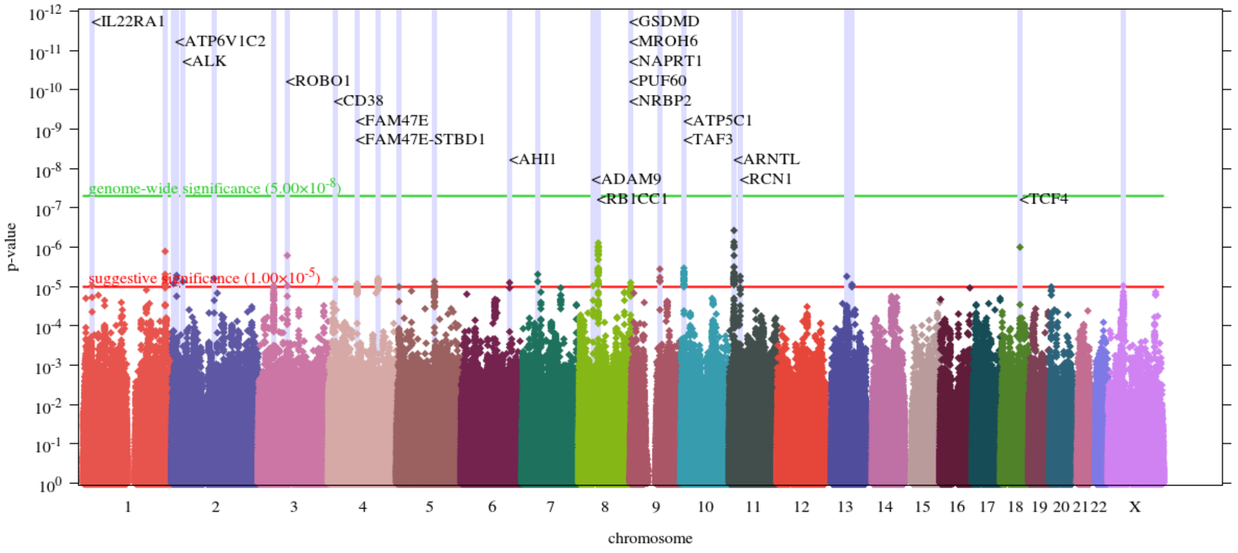

- **Supplementary Figure S1d**. SNP level Manhattan plots for GWAS analysis - high TLR subgroup.

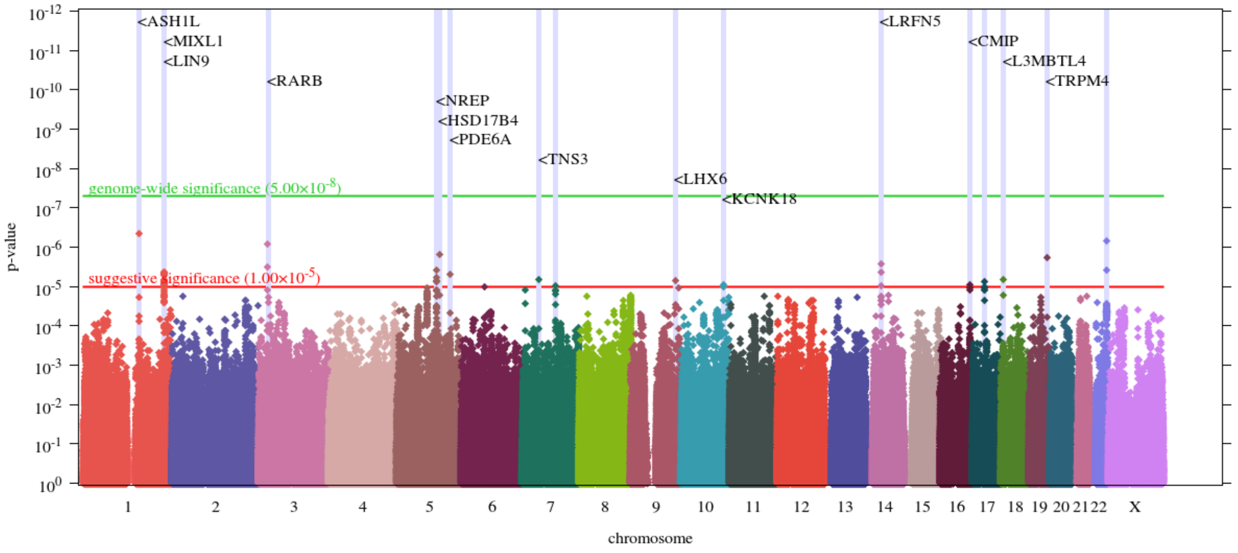

- **Supplementary Figure S1e**. SNP level Manhattan plots for GWAS analysis - total population.

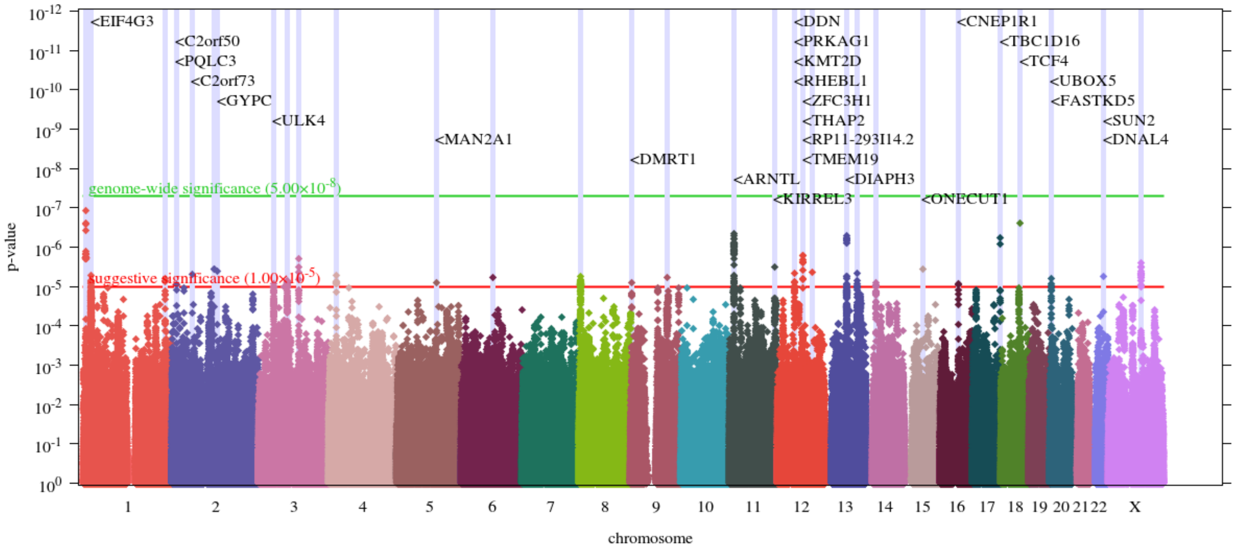

<EIF4G3
<C2orf50
<PQLC3
<C2orf73
<GYPC
<ULK4
<MAN2A1
<DMRT1
<ARNTL
<KIRREL3
<DDN
<PRKAG1
<KMT2D
<RHEBL1
<ZFC3H1
<THAP2
<RP11-293I14.2
<TMEM19
<DIAPH3
<ONECUT1
<CNEP1R1
<TBC1D16
<TCF4
<UBOX5
<FASTKD5
<SUN2
<DNAL4
genome-wide significance (5.00×10^-8)
suggestive significance (1.00×10^-5)
p-value
chromosome
1 2 3 4 5 6 7 8 9 10 11 12 13 14 15 16 17 18 19 20 21 22 X

- **Supplementary Figure S2a.** Comparison between gene level results of the low TLR subgroup and the total population. Kynurenine and serotonin pathway-related genes are denoted with blue and red markers respectively, other SNPs are denoted with grey markers.

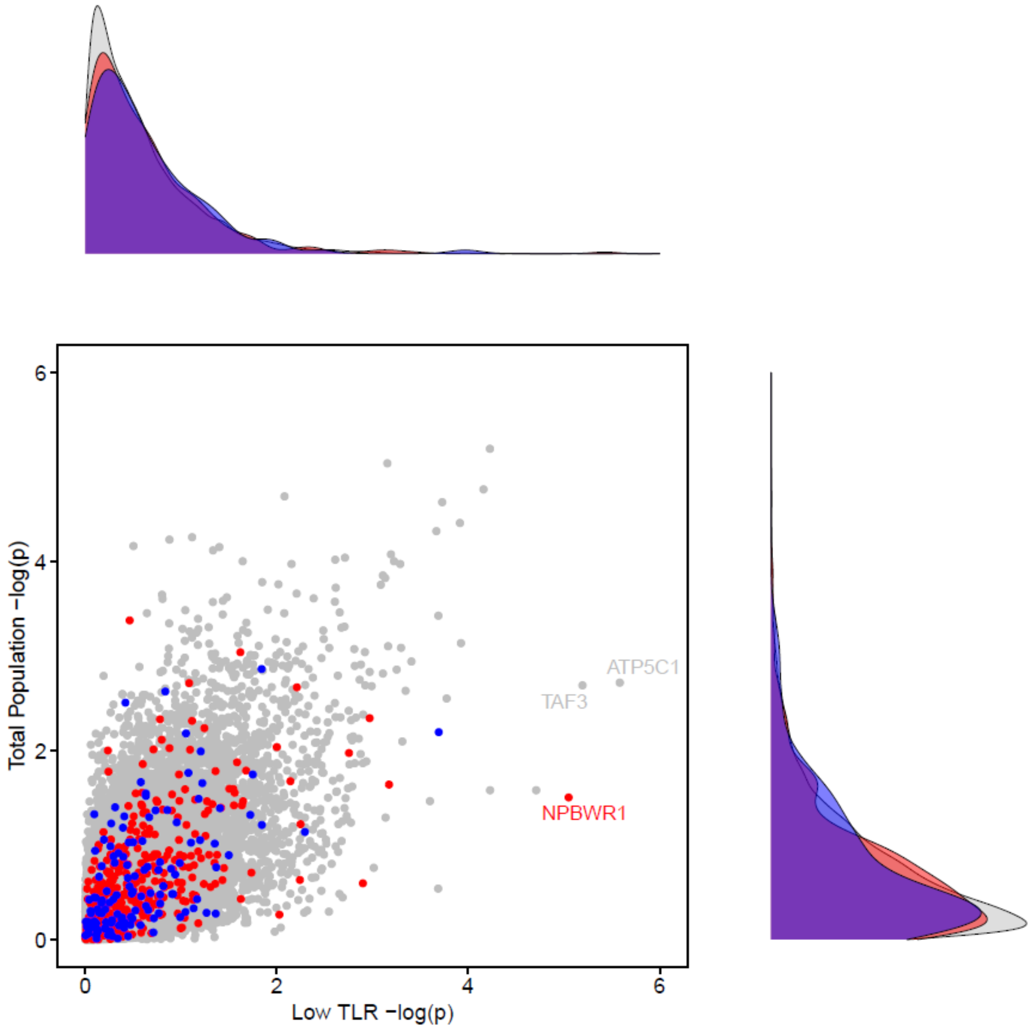

- **Supplementary Figure S2b.** Comparison between gene level results of the high TLR subgroup and the total population. Kynurenine and serotonin pathway-related genes are denoted with blue and red markers respectively, other SNPs are denoted with grey markers.

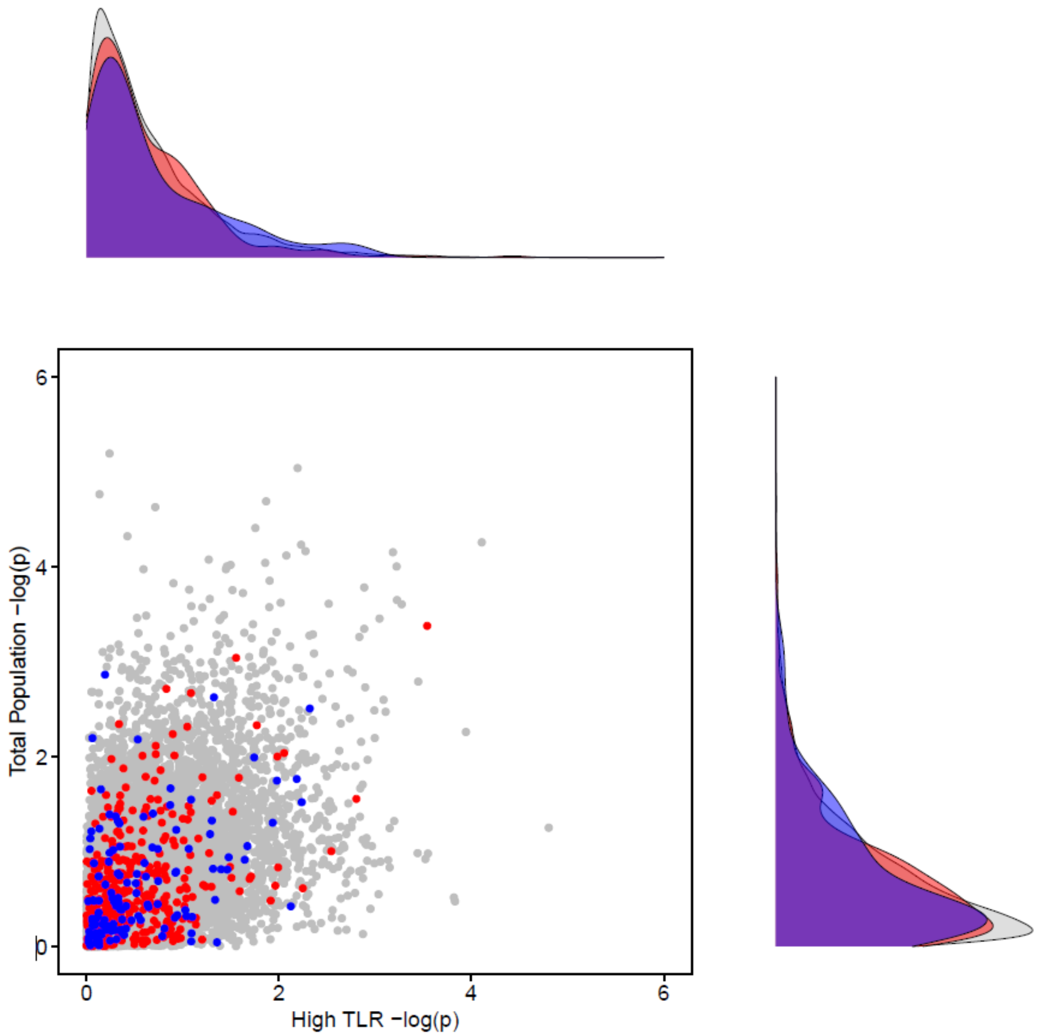

- **Supplementary Figure S3a.** Pathway level associations with respect to depressive symptoms for candidate serotonin pathways, kynurenine pathways, and GO terms for the low TLR subgroup versus total population. P-values are displayed with correction for set size using a regression based method in the form of -log10 p.

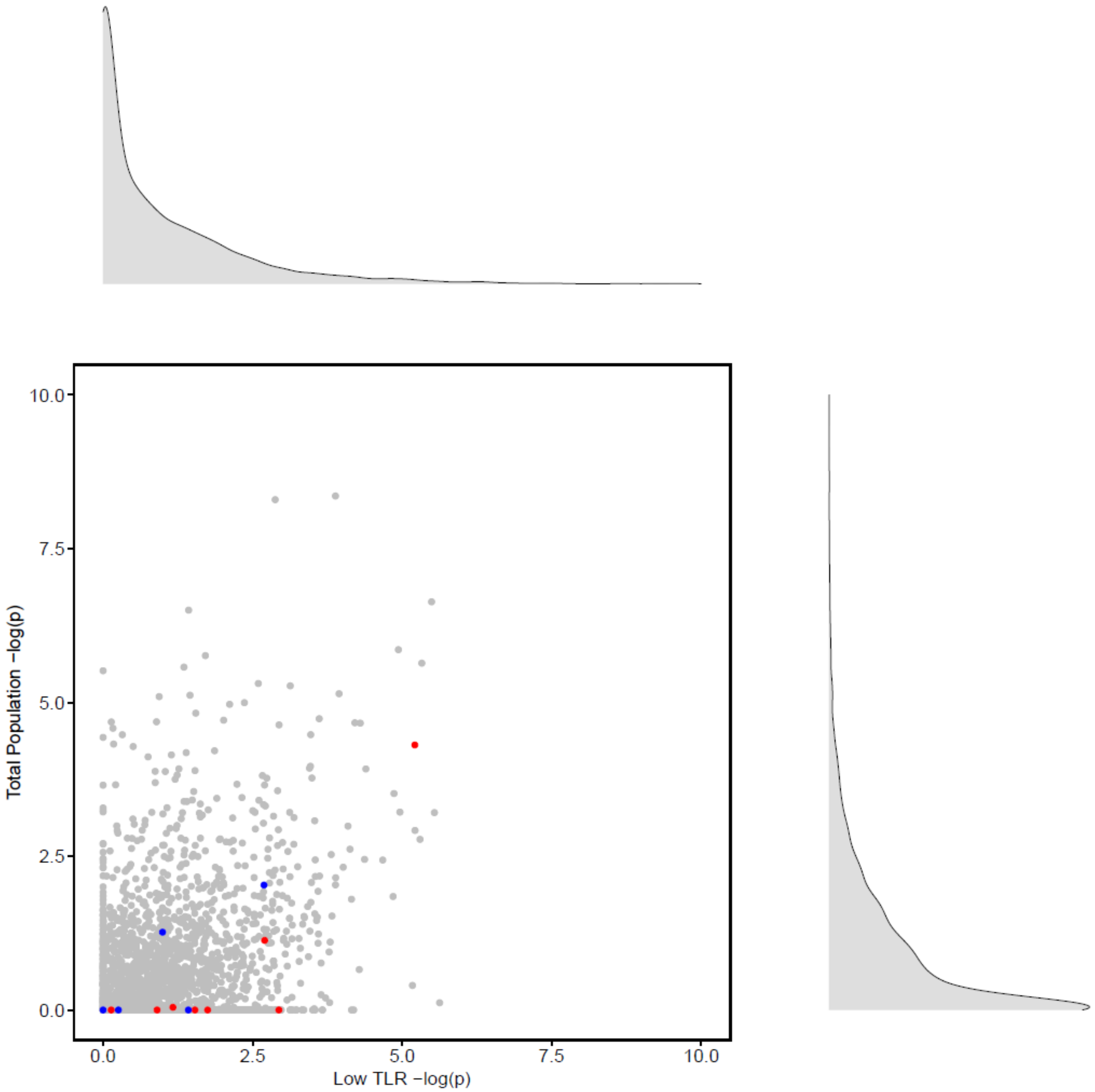

- **Supplementary Figure S3b.** Pathway level associations with respect to depressive symptoms for candidate serotonin pathways, kynurenine pathways, and GO terms for the high TLR subgroup versus total population. P-values are displayed with correction for set size using a regression-based method in the form of -log10 p.

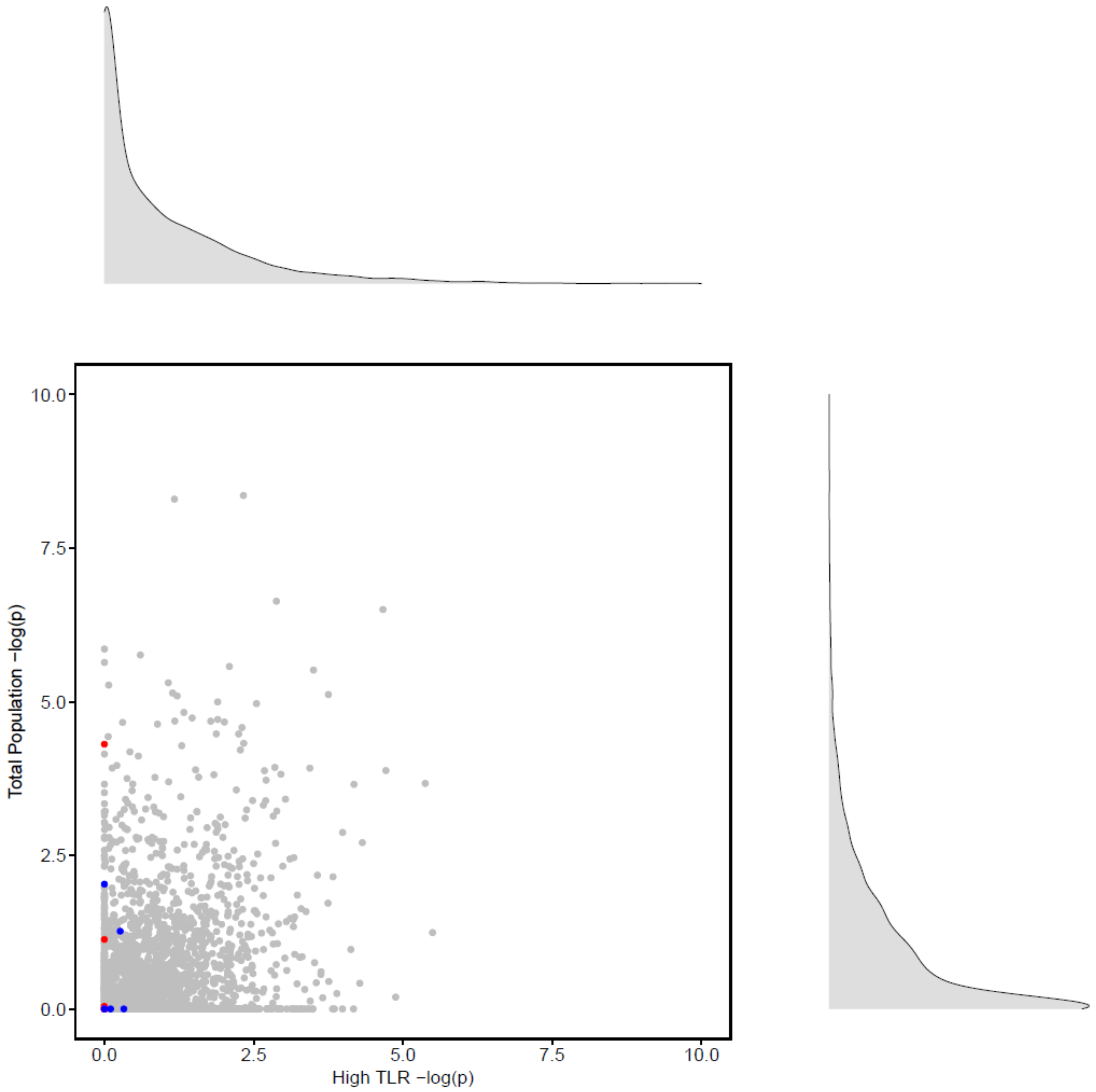

- **Supplementary Figure S4a.** Pathway level associations with respect to depressive symptoms for candidate serotonin pathways, kynurenine pathways, and GO terms for the low TLR versus the high TLR subgroup. P-values are displayed without correction for set size in the form of -log10 p.

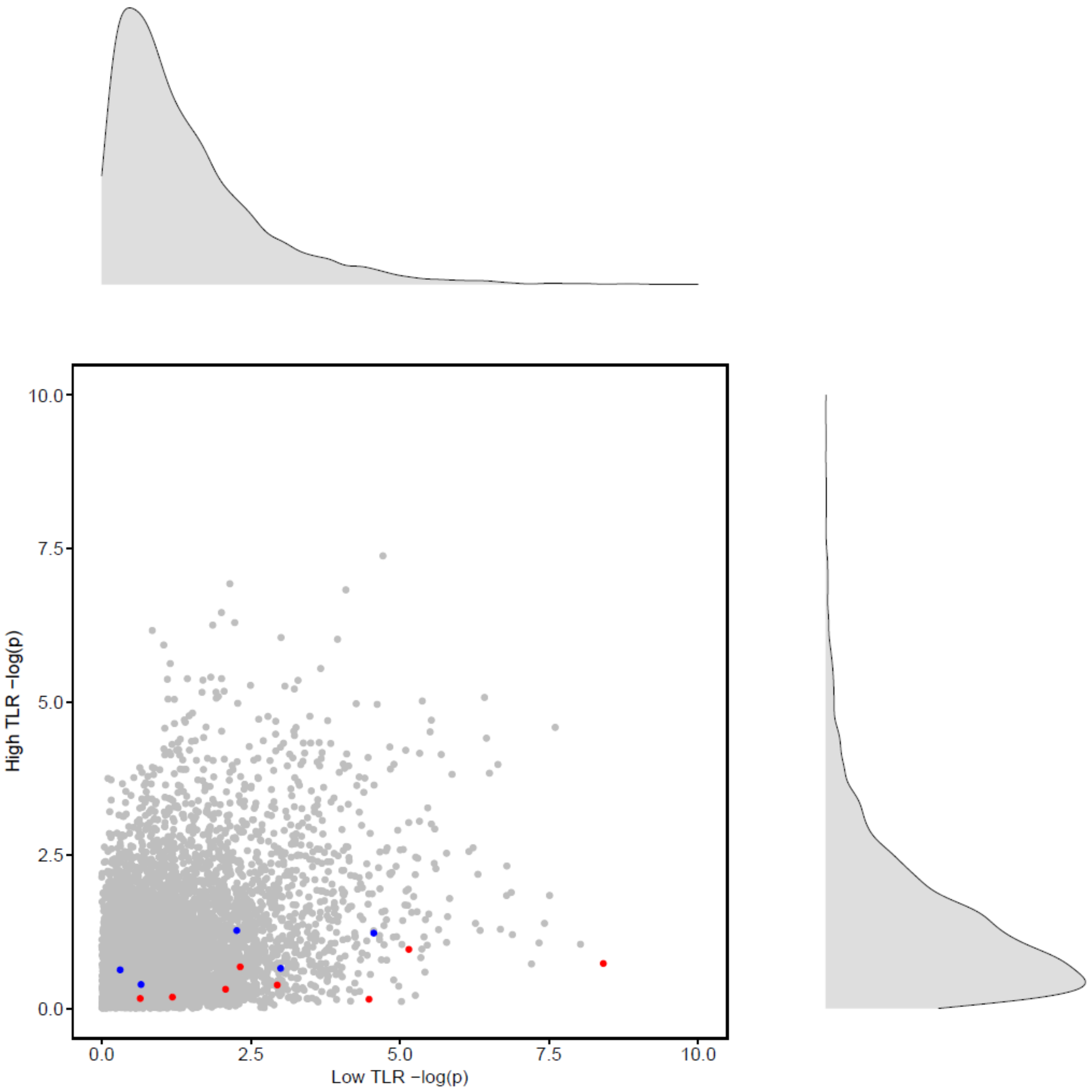

- **Supplementary Figure S4b.** Pathway level associations with respect to depressive symptoms for candidate serotonin pathways, kynurenine pathways, and GO terms for the low TLR subgroup versus total population. P-values are displayed without correction for set size in the form of -log10 p.

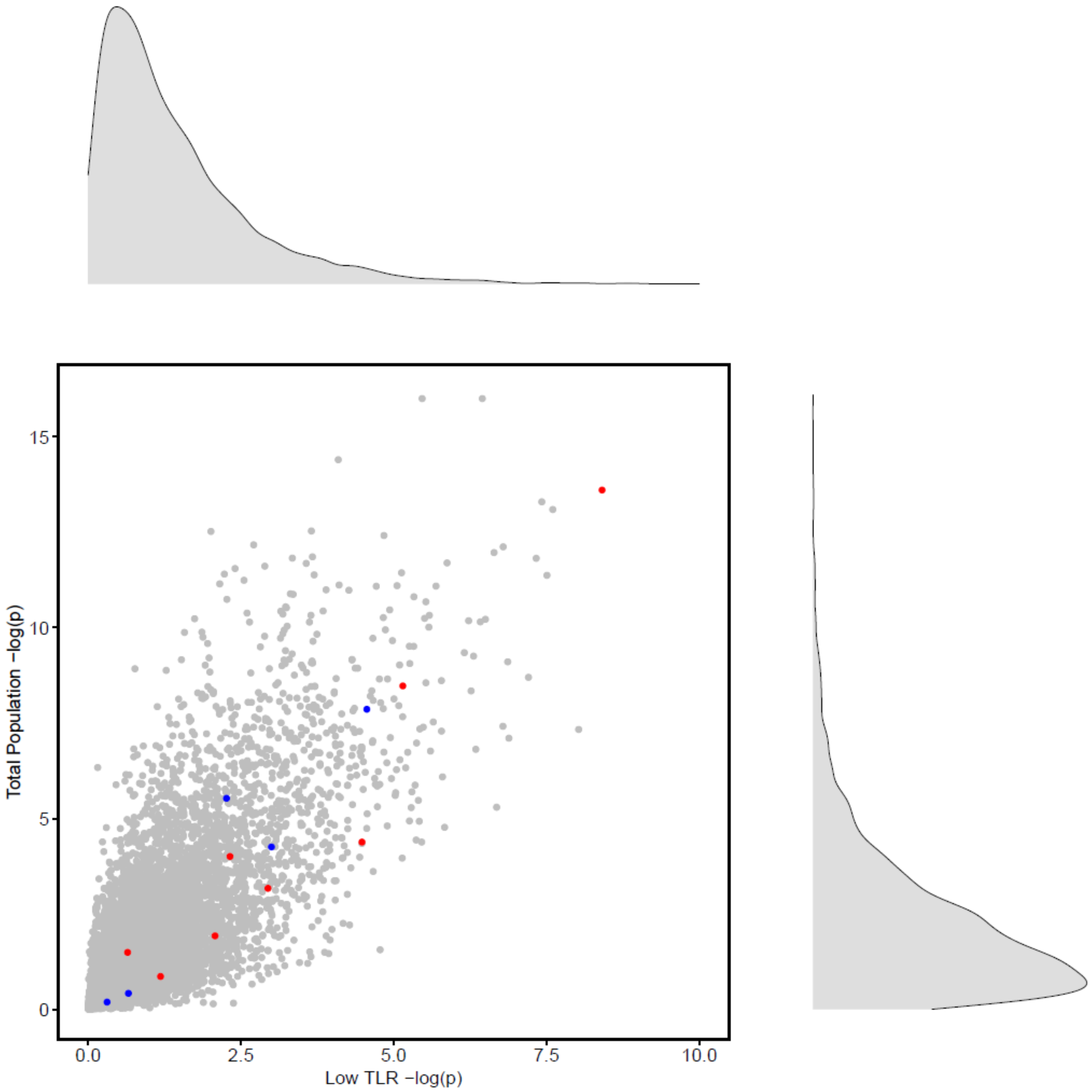

- **Supplementary Figure S4c.** Pathway level associations with respect to depressive symptoms for candidate serotonin pathways, kynurenine pathways, and GO terms for the high TLR subgroup versus total population. P-values are displayed without correction for set size in the form of –log10 p.

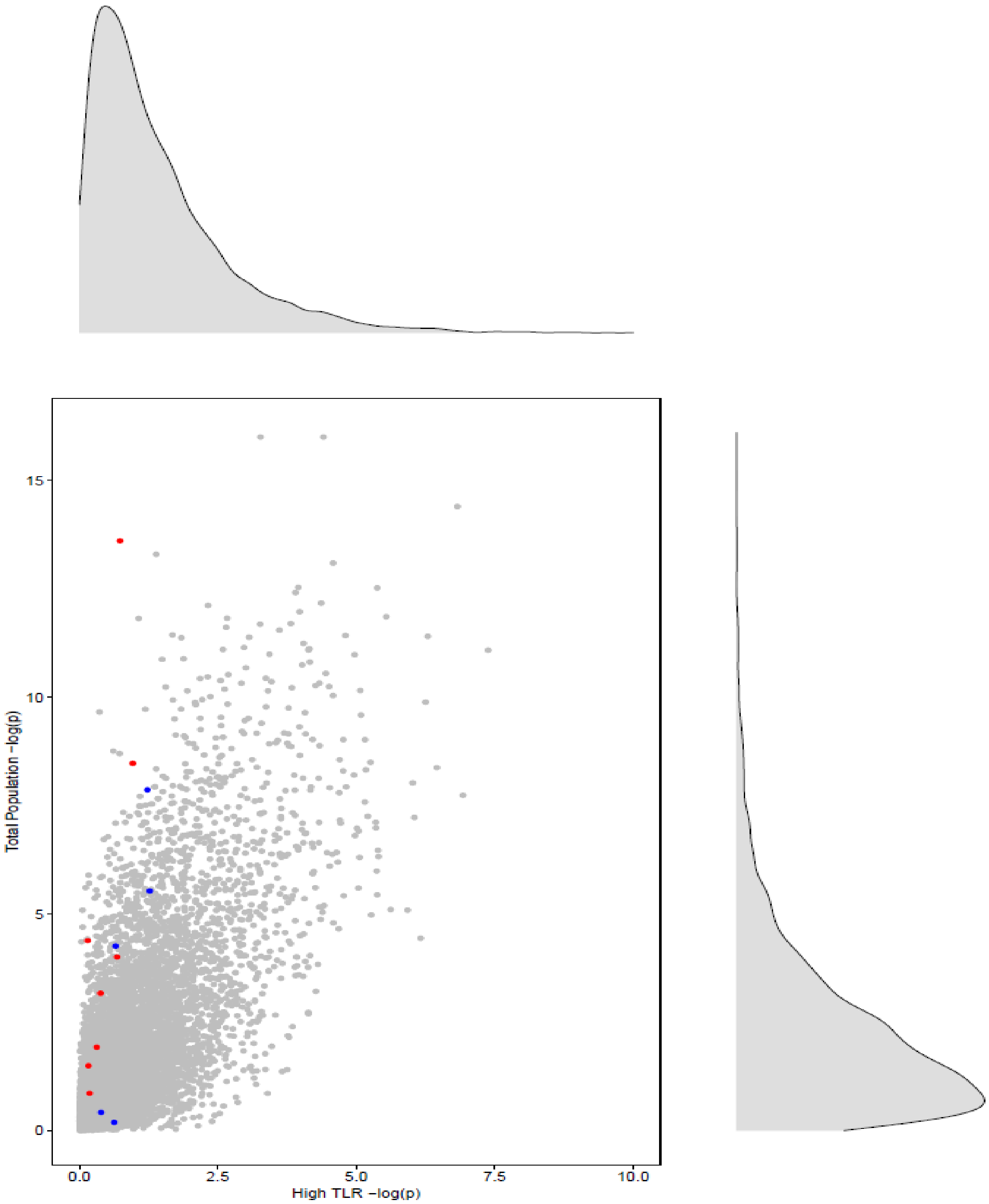

- **Supplementary Figure S5**. The steps for quality control of genetic data following the UK biobank QC guidelines. For further description see section 2.2 in Supplementary materials.

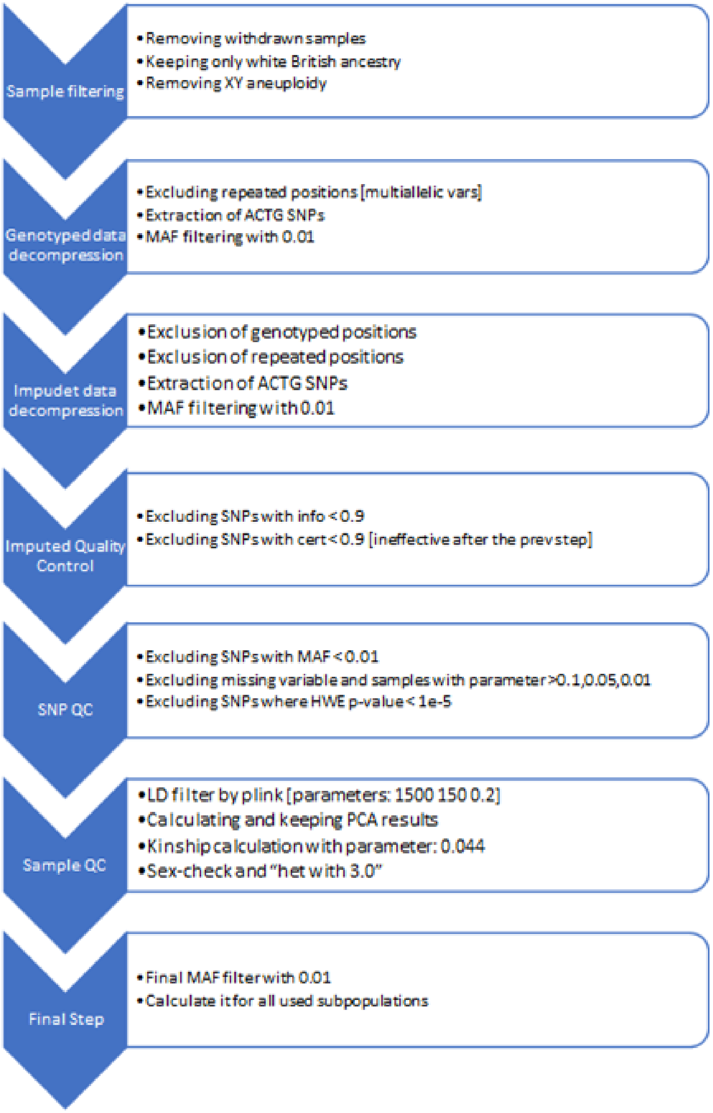

- **Supplementary Figure S6a. Serotonin gene set sources.** Set sizes visually depicted by circles correspond to the number of genes in that set. Colouring corresponds to a given database source in which that set of genes is related to the **serotonin pathway**. In cases when a set of genes can be found in multiple sources, those sets are depicted as part of multiple coloured fields. Genes related to (1) G-protein coupled receptor downstream signalling in the Reactome database (Reactome_down) are displayed in red, (2) serotonin receptors in the Reactome database (Reactome_serot) are displayed in green, (3) combined Gene Ontology (GO) terms (selected and merged as described in methods) are displayed in yellow, (4) WikiPathway (WP) terms (processed according to methods) are displayed in blue.

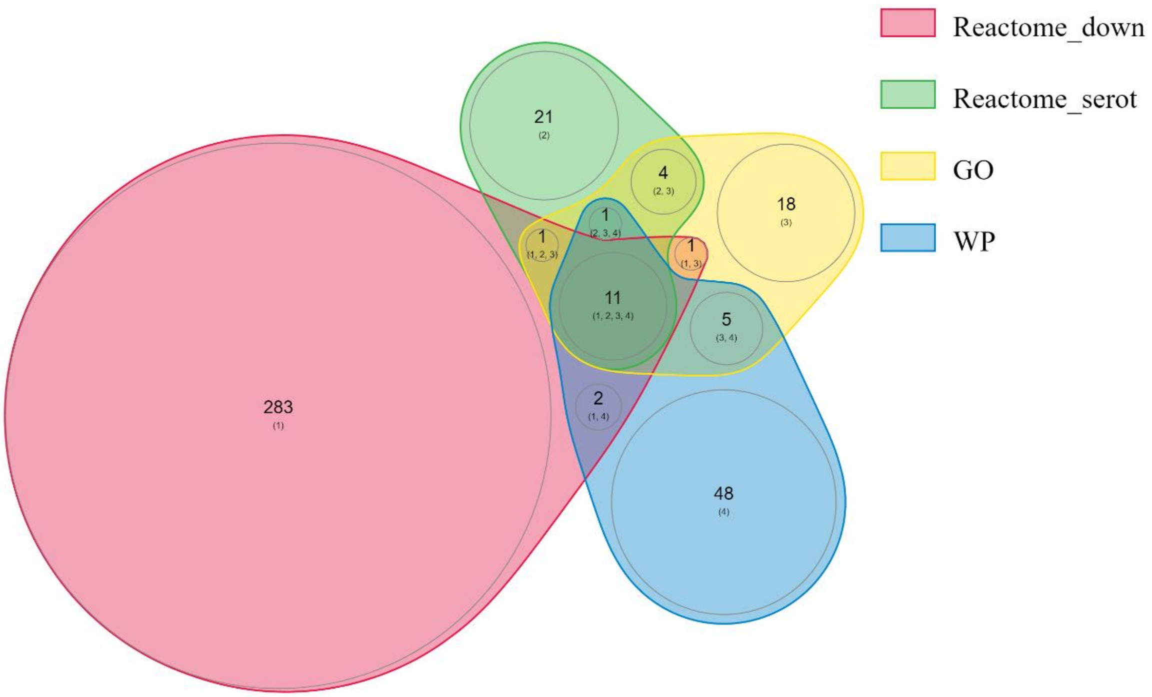

- **Supplementary Figure S6b. Kynurenine gene set sources**. Set sizes visually depicted by circles correspond to the number of genes in that set. Colouring corresponds to a given database source in which that set of genes is related to the **kynurenine pathway**. In cases when a set of genes can be found in multiple sources, those sets are depicted as part of multiple coloured fields. Genes related to (1) non-specific binders of kynurenine pathway metabolites based on the Chembl database (Chembl) are displayed in red, (2) combined Gene Ontology (GO) terms (selected and merged as described in methods) are displayed in green, (3) WikiPathway (WP) terms (processed according to methods) are displayed in yellow, (4) kynurenine metabolic pathway in the Reactome database (Reactome) are displayed in blue.

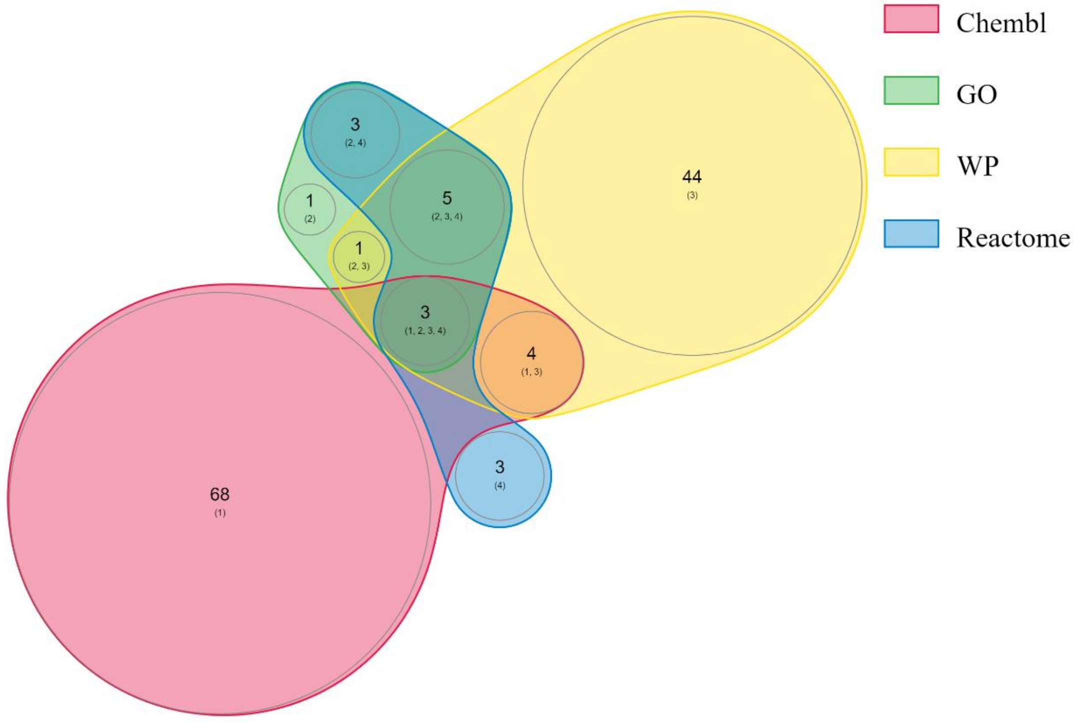

2. Additional information on methods

2.1 Derivation of TLR

Assessing the protein content of various food types, more specifically their amino acid content, was the first step of computing TLR, i.e. tryptophan - large neutral amino acid (LNAA) ratio of a given diet. The National Diet and Nutrition survey (NDNS, https://www.gov.uk/government/statistics/ndns-results-from-years-5-and-6-combined)[1] was utilized to determine which foods contribute most in terms of intake of various amino acids. Relying on NDNS the top 20 food sources were identified taking into consideration which foods were consumed the most often and in the highest quantities to influence amino acid intake. This included the assessment of average daily consumption in grams of each food and the percentage contribution of each food to protein intake. Such an analysis was carried out for tryptophan and for the other large neutral amino acids: valine, isoleucine, leucine, tyrosine, phenylalanine [2]. Each relevant NDNS food category was matched to UK biobank food categories. Amino acid content for each selected food (per portion) was then calculated using the United States Department of Agriculture (USDA) Food Composition Database (https://www.nal.usda.gov/usda-food-composition-database) [3]. Portion sizes were based on the Food Portion sizes handbook [4] of the Ministry of Agriculture, Fisheries and Food (MAFF). Based on the reported diet, i.e. food type and quantity, the tryptophan and LNAA intake was estimated and their ratio (TLR) was computed for each subject. Altogether five measurement instances were available in the UKB data set for dietary variables. Some subjects were queried in all five instances, some were queried only once. Finally, computed TLR values were averaged over available instances for each subject.

2.2 QC methodology

The following QC steps were performed on the whole dataset. First, we removed samples the consent of which were withdrawn, were not of white British ancestry, or putatively had a sex chromosome aneuploidy (see Supplementary Figure S5). Second, only biallelic SNP variants were retained, from both the set of genotyped and imputed variants, furthermore, imputed variants with ‘info’ or ‘certainty’ values lower than 0.9 were also excluded.

Genotyped and imputed genotypic values were merged, with genotyped ones having precedence. To exclude samples and variants which might be considered unreliable for the purpose of statistical tests, the following filtering steps were performed:

(1) variants with minor allele frequency values lower than 0.01 were excluded
(2) variants and samples with missing rates greater than 0.01 were excluded (this was done in an iterative manner, with more and more rigorous threshold values 0.1, 0.05 and 0.01, the filters alternatingly applied first to variants then to samples for each threshold value)
(3) variants which could be assumed not to be in Hardy-Weinberg equilibrium with a p-value more relevant than (i.e. less than) 1e-5 were excluded

The set of variants that was achieved after the above steps was regarded as the input of our association tests.

Samples were further filtered using the following steps:

(4) Samples whose F-statistics (as calculated by the command '--check-sex' by plink) were not unambiguous were removed.
(5) Samples which were deemed outliers regarding their observed and expected autosomal homozygous genotype counts were removed.
(6) Finally, variants with a minor allele frequency less than 0.01 in either of the subpopulations were excluded.

Note: The QC used in BOLT-LMM and set-test does not include steps for kinship filtering because of these methods can handle related samples, hence there is less information loss in the process.

3. Additional results

3.1 Relevant serotonin and kynurenine SNPs

The serotonin SNP rs10089380 located in the 10 kilobase neighbourhood of the *RB1CC1* gene (RB1 inducible coiled-coil 1) is one among the suggestively significant (p=1.6E-06) SNPs. In addition, this SNP is also part of the regulatory region of *NPBWR1* (neuropeptides B and W receptor 1). Furthermore, 35 SNPs between positions 53669625 and 53823272 with suggestive significance also belong to the same regulatory region of *NPBWR1* according to GeneHancer. Note

that none of these SNPs are significant with respect to the serotonin pathway level corrected significance threshold of 1.68E-07.

The kynurenine SNP rs4471056 is a downstream variant situated near *MROH6* (maestro heat like repeat family member 6) at position 144647964, but it is also in the 10 kilobase neighbourhood of *GSDMD* (gasdermin D), *NAPRT1* (nicotinate phosphoribosyltransferase), and *AC067930.4*. In addition, this SNP is also among the eQTL of *TSTA3* (tissue specific transplantation antigen P35B), and of *TIGD5* (tigger transposable element derived 5). The other SNP rs149007785 is a transcript variant of *NRBP2* (nuclear receptor binding protein 2), and it is also in the 10 kilobase neighbourhood of *PUF60* (poly(U) binding splicing factor 60), *AC234917.1*, and it is also among the eQTL of *ZNF623, AC067930.4, NAPRT1, ZFP41*, and *MROH6*. Note that these SNPs are not significant with respect to the kynurenine pathway level corrected significance threshold of 4.24E-07.

3.2 Relevant SNPs not related to candidate pathways

Regarding SNPs outside of our candidate pathways, in the low TLR subgroup exclusively, 119 SNPs showed association with depressive symptoms with suggestive significance (p<1E-05). Some of these SNPs are intergenic (76), others (43) are related to the following genes: *IL22RA, ATP6V1C2, ALK, ROBO1, CD38, FAM47E, AHI1, ADAM9, ATP5C1, TAF3, ARNTL, RCN1, TCF4.* For details see **Supplementary Table S1**. In the high TLR subgroup 40 SNPs showed association with depressive symptoms with suggestive significance. Four SNPs are intergenic, the other 36 SNPs are related to the following genes: *ASH1L, LIN9, RARB, NREP, HSD17B4, PDE6A, TNS3, LHX6, KCNK18, LRFN5, CMIP, L3MBTL4, TRPM4*. For details see **Supplementary Table S2**. Note that there is no intersection between the two sets of suggestively significant SNPs corresponding to low and high TLR subpopulations.

Regarding the total population, 246 SNPs showed association with depressive symptoms with suggestive significance. 58 SNPs are related to the following genes: *EIF4G3, C2orf50, C2orf73, GYPC, ULK4, MAN2A1, DMRT1, ARNTL, KIRREL3, DDN, KMT2D, RP11-293I14.2, DIAPH3, CNEP1R1, TBC1D16, TCF4, DNAL4*, and 188 SNPs are intergenic. For details see **Supplementary Table S3.** Note that 4 SNPs in *ARNTL* (rs10832027, rs10766078, rs12421530, rs3816360), rs10503002 in *TCF4,* and 41 intergenic SNPs are suggestively significantly associated

with depressive symptoms in both the low TLR subgroup and the total population. Further association results with respect to depressive symptoms are provided for serotonin SNPs (**Supplementary Table S15)**, kynurenine SNPs (**Supplementary Table S16**) and for all other SNPs (**Supplementary Table S17**) in Supplementary materials.

3.3 Relevant serotonin and kynurenine genes: *NPBWR1* and *POLI*

NPBWR1 shows the highest expression levels in blood, liver and brain, especially in the cerebral cortex, hypothalamus and pituitary gland, according to the Human Protein Atlas v20.0 [5]. Neuropeptide W and neuropeptide B, ligands of the G protein-coupled receptor encoded by this gene, play a role in neuroendocrine regulation, energy homeostasis, feeding behaviour, circadian rhythm and sleep, emotions, as well as in pain sensation and cardiovascular functions (Chottova Dvorakova, 2018). In animal studies, *NPBWR1* has been associated with stress response in new environments [6], with social behaviour and fear processing [7], and with metabolic disturbances and adult-onset obesity [8], which traits are also highly related to depression. In human studies, polymorphisms in the *NPBWR1* gene altered face emotion processing [9], and in GWAS studies were associated with obesity-related variables [10,11], age at menarche [12], and response to antidepressant [13]. Thus, based on previous studies and our present results, *NPBWR1* may be a good target for novel treatment strategies in antidepressive therapies.

In the kynurenine pathway, the canonical candidate genes encoding the rate limiting metabolic enzymes of tryptophan catabolism – the indoleamine 2,3-dioxygenase (IDO) and tryptophan 2,3-dioxygenase (TDO) [14] – did not emerge as top genes for depressive symptoms. The only significant association in the low TLR subgroup was with the *POLI* (DNA polymerase iota) gene, which only survived kynurenine pathway-level but not all gene-level correction. There were no kynurenine associations in the high TRL subgroup or in the total cohort. POLI is connected to the kynurenine pathway via potential binding with kynurenine metabolites (3-Hydroxyanthranilic Acid and Quinolinic Acid) and is mainly expressed in endocrine tissues [5], including the testis and ovary, and the brain, especially in the cortex, the pons and medulla. Its main function is to promote DNA repair. Interestingly, a recent multivariate genome-wide-association meta-analysis study

focusing on well-being-related traits, such as life satisfaction, positive affect, neuroticism, and depressive symptoms, demonstrated a significant association with regulatory regions of *POLI* [15].

3.4 Relevant genes not related to candidate pathways

Amongst all genes, *ATP5C1*, *TAF3* showed significant and the *KIN* gene suggestive significant association with depressive symptoms in the low TLR subgroup (see **Supplementary Table S6**). Furthermore, 20 genes showed association with suggestive significance in the total population (*IFNLR1*, *ZFC3H1*, *IL22RA1*, *DIAPH3*, *UBOX5*, *ROBO1*, *MYOM3*, *DHH*, *EIF4E3*, *DCLK1*, *LMBR1L*, *EP300*, *TCF4*, *PPME1*, *SLC4A11*, *GYPC*, *RHEBL1*, *CTNNB1*, *RCN1*, *NUMB*) (see **Supplementary Table S6**).

In addition, gene-level results were compared between the total population and TLR subgroups (see **Supplementary Figure 2a** for comparison between low TLR subgroup and the total population, and **Supplementary Figure 2b** for high TLR subgroup and the total population results). Both supplementary figures show that none of the serotonin or kynurenine-related genes were significantly associated with depressive symptoms in the total population.

3.5 Relevant pathways

Results related to the subsets of kynurenine pathway indicated that the non-specific binders subset from CHEMBL was associated with depressive symptoms in the low TLR subgroup and in the total population with p-value: 0.017 and 0.0034 respectively, which however was not significant (using correction for kynurenine subpathways, p-value<0.0033). In addition, the kynurenine WikiPathway subset (denoted as Kynurenine WP) showed significant association with depressive symptoms in the total population with p-value: 4.7E-4.

For the serotonin set, subpathways from all main sources (WikiPathway, GO and Reactome) showed associations with depressive symptoms in the low TLR subgroup and also in the total population. The Reactome serotonin subpathway showed a significant association both in the low TLR subgroup (p-value: 0.0015) and in the total population (p-value: 0.0016) that survived correction for serotonin subpathways (p-value<0.0021). Furthermore, the Serotonin WikiPathway only showed a significant association in the low TLR subgroup (p-value: 0.0019), whereas Reactome GPCR downstream and Serotonin GO showed a significant association only in the total

population (p-value 0.0013). Importantly, none of the main subpathways was significant in the high TLR subgroup (for details see **Supplementary Table S7**).

3.6 Relevant GO terms

In the whole population, the most significant pathway was "adult behaviour" and several pathways related to brain development. In the low TLR subgroup, the most significant pathways associated with depression included "adaptive thermogenesis" (GO:1990845), "cerebral cortex cell migration" (GO:0021795), "positive regulation of cold induced thermogenesis" (GO:0120162), "negative regulation of ERBB signalling pathway" (GO:1901185) and "regulation of neural precursor cell proliferation" (GO:2000177), while "blood vessel endothelial cell migration" (GO:0043534) and "response to organophosphorus" (GO:0046683) were also significant in the whole population. In contrast, in the high TLR subgroup only "regulation of calcium ion transmembrane transport" (GO:1903169) and "negative regulation of peptidyl serine phosphorylation" (GO:0033137) emerged as significant mechanisms with no overlap with significant pathways with either the low TLR or the total population.

Focusing on mechanisms significantly associated with depression in the low dietary TLR subgroup, "adaptive thermogenesis" pathway (GO:1990845) is related to regulated heat production in response to short-term environmental changes including for example stress, diet, or reduced temperature, and is implicated in reduced body weight maintenance and unsuccessful weight loss [16]. Thus adaptive thermogenesis may be clinically relevant with respect not only to diet but also depression, considering that depression is a stress-related condition associated with weight changes in either direction, which, especially weight gain, may play a crucial role in the negative consequences of depression on somatic health especially increased cardiovascular risk [17,18] and metabolic comorbidities in depression [19]. Several of the other most significant pathways in the low dietary TLR subgroup reflect aspects of neurodevelopment. The "cerebral cortex cell migration pathway" (GO:0021795), controlling key morphogenic events involved in neocortical neurodevelopment contributing to a precise spatial and temporal distribution of neural cells and also their maturation and formation of neural circuits has been implicated in neuropsychiatric disorders including first episode psychosis or schizophrenia [20], susceptibility to juvenile myoclonic epilepsy [21], or autism spectrum disorder associated with 16p11.2 copy number variation [22]. "Negative regulation of ERBB signalling pathway" (GO:1901185) via neuregulin/ERBB

signalling also plays a role in neurodevelopment via regulating the assembly of neural circuitry playing a role also in synaptic plasticity, myelination and neurotransmission, with abnormal signalling contributing to impaired brain function and to susceptibility of neuropsychiatric disorders including depression, bipolar disorder or schizophrenia [23]. Besides multiple and in part overlapping pathways and mechanisms significantly associated with depression in the low dietary TLR subgroup and the total population, in the high dietary TLR subgroup there was a remarkable relative lack of significantly associated pathways, with only general and not solely brain-relevant mechanisms such as "regulation of calcium ion membrane transport" (GO:1903169) and "negative regulation of peptidyl serine phosphorylation" (GO:0033137) showing an association, and none of the mechanisms that showed association in the low dietary TLR subgroup or the whole sample were significant in the high TLR subgroup.

Besides "cerebral cortex cell migration pathway" and "negative regulation of ERBB signalling pathway", the GO term "regulation of neural precursor cell proliferation" (GO:2000177) is also associated with brain development. Neural precursor cells, including neural stem cells and neural progenitor cells, are temporally regulated cells with the ability for generating all neural cell types present in the brain via proliferation and differentiation into astrocytes, oligodendrocytes and mature neurons in the presence of growth factors. Especially adult neurogenesis has attracted significant attention as implicated in human neuropsychiatric disorders and also possibly in their treatment, although results are conflicting [24-27]. Considering that mechanisms related to brain development were significant only on the low TLR subgroup or the total sample but not the high TLR subgroup, our results suggest that the effect of such risk variants may be a function of diet, keeping in mind that several of the above processes reflect early neurodevelopment unaffected by current dietary habits. Interestingly, the most significant pathways in the whole population (in addition to being significant also in the low TRL subgroup) included "Blood vessel endothelial cell migration" (GO:0043534), related to angiogenesis which may be relevant in several ways. On the one hand, considering that blood vessels constitute key components of stem cell niches also in the adult brain controlling neuronal stem cell function [28], this finding further supports the suggestion that TLR intake may be associated with depression via influencing neurogenesis. Furthermore, blood vessel endothelial cell migration may also be related to cardiovascular risk and thus be important in the context of the increased cardiovascular comorbidity and mortality observable in depression [17,18]. The other most significant pathway in the total population was

"response to organophosphorous" (GO:0046683) which may have relevance with respect to dementia, considering the role of organophosphate acetylcholinesterase inhibitors employed in the treatment of Alzheimer's disease and other dementias, and also the association with depression with earlier onset of cognitive decline [29].

4. Addition to the rational of the study: effects of acute tryptophan depletion challenge in human and animal studies

Previous studies demonstrated that ATD induces emotional, cognitive and memory abnormalities only in individuals with susceptibility to depressive and anxiety disorders[30-32] and had no or minimal effect on mood in healthy individuals without family history for psychiatric disorders [33]. Furthermore, animal studies showed that after ATD the decrease in 5-HT synthesis is not ubiquitous in the brain, it is more effective in the hippocampus, but also relevant in frontal and prefrontal cortex. In addition, these effects were more prominent in mice strain with impaired 5-HT synthesis and a more anxious phenotype[34]. In humans, ATD has not changed the default mode network (DMN) connectivity in healthy volunteers, despite that plasma TRP level decreased significantly, which is especially interesting as DMN connectivity was associated with depressive symptoms[35]. Therefore, in this study, our aim was to provide explanation for differential susceptibility to the development of depressive symptoms in those with habitual low dietary ratio of TRP/LNAA and to identify which genetic risk factors contribute to depression in case of low or high dietary ratio of TRP/LNAA. Based on previous negative depression GWAS studies regarding the classical canonical serotonin and kynurenine related genes we would expect perturbation of the downstream signalling mechanisms and regulatory biological processes of these candidate pathways.

References

1 Agency, P. H. E. a. t. F. S. National Diet and Nutrition Survey. Results from Years 5-6 (combined) of the Rolling Programme (2012/13 –2013/14). (2016).

2 Fernstrom, J. D. Large neutral amino acids: dietary effects on brain neurochemistry and function. *Amino Acids* **45**, 419-430, doi:10.1007/s00726-012-1330-y (2013).
3 (USDA), U. S. D. o. A. (2017).
4 Agency, F. S. *Food Portion Sizes (Maff Handbook)*. (The Stationary Office, 2002).
5 Uhlen, M. *et al.* Proteomics. Tissue-based map of the human proteome. *Science* **347**, 1260419, doi:10.1126/science.1260419 (2015).
6 Motoike, T. *et al.* Mesolimbic neuropeptide W coordinates stress responses under novel environments. *Proc Natl Acad Sci U S A* **113**, 6023-6028, doi:10.1073/pnas.1518658113 (2016).
7 Nagata-Kuroiwa, R. *et al.* Critical role of neuropeptides B/W receptor 1 signaling in social behavior and fear memory. *PLoS One* **6**, e16972, doi:10.1371/journal.pone.0016972 (2011).
8 Ishii, M., Fei, H., Friedman, J.M. . Targeted disruption of GPR7, the endogenous receptor for neuropeptides B and W, leads to metabolic defects and adult-onset obesity. *Proc Natl Acad Sci U S A* **100**, 10540-10545 (2003).
9 Watanabe, N., Wada, M., Irukayama-Tomobe, Y., Ogata, Y., Tsujino, N., Suzuki, M., Furutani, N., Sakurai, T., Yamamoto, M. A single nucleotide polymorphism of the neuropeptide B/W receptor-1 gene influences the evaluation of facial expressions. *PLoS One* **7**, e35390 (2012).
10 Richardson, T. G., Sanderson, E., Elsworth, B., Tilling, K. & Davey Smith, G. Use of genetic variation to separate the effects of early and later life adiposity on disease risk: mendelian randomisation study. *BMJ* **369**, m1203, doi:10.1136/bmj.m1203 (2020).
11 Tachmazidou, I. *et al.* Whole-Genome Sequencing Coupled to Imputation Discovers Genetic Signals for Anthropometric Traits. *Am J Hum Genet* **100**, 865-884, doi:10.1016/j.ajhg.2017.04.014 (2017).
12 Perry, J. R. *et al.* Parent-of-origin-specific allelic associations among 106 genomic loci for age at menarche. *Nature* **514**, 92-97, doi:10.1038/nature13545 (2014).
13 Fabbri, C. *et al.* New insights into the pharmacogenomics of antidepressant response from the GENDEP and STAR*D studies: rare variant analysis and high-density imputation. *Pharmacogenomics J* **18**, 413-421, doi:10.1038/tpj.2017.44 (2018).
14 Boros, F. A., Bohar, Z. & Vecsei, L. Genetic alterations affecting the genes encoding the enzymes of the kynurenine pathway and their association with human diseases. *Mutat Res* **776**, 32-45, doi:10.1016/j.mrrev.2018.03.001 (2018).
15 Baselmans, B. M. L. *et al.* Multivariate genome-wide analyses of the well-being spectrum. *Nat Genet* **51**, 445-451, doi:10.1038/s41588-018-0320-8 (2019).
16 Major, G. C., Doucet, E., Trayhurn, P., Astrup, A. & Tremblay, A. Clinical significance of adaptive thermogenesis. *Int J Obes (Lond)* **31**, 204-212, doi:10.1038/sj.ijo.0803523 (2007).
17 Gan, Y. *et al.* Depression and the risk of coronary heart disease: a meta-analysis of prospective cohort studies. *BMC Psychiatry* **14**, 371, doi:10.1186/s12888-014-0371-z (2014).
18 Wu, Q. & Kling, J. M. Depression and the Risk of Myocardial Infarction and Coronary Death: A Meta-Analysis of Prospective Cohort Studies. *Medicine (Baltimore)* **95**, e2815, doi:10.1097/MD.0000000000002815 (2016).
19 Marazziti, D., Rutigliano, G., Baroni, S., Landi, P. & Dell'Osso, L. Metabolic syndrome and major depression. *CNS Spectr* **19**, 293-304, doi:10.1017/S1092852913000667 (2014).
20 Maes, M., Plaimas, K., Suratanee, A., Noto, C. & Kanchanatawan, B. The Protein-Protein Interaction Network of First Episode Psychosis and Schizophrenia Reveals Possible Trigger Factors and New Drug Targets among Intracellular Signal Transduction Pathways and Neurotoxicity Processes. *Preprints*, doi:10.20944/preprints202107.0596.v1 (2021).

21 Ganesh, S. *et al.* Exome sequencing in families with severe mental illness identifies novel and rare variants in genes implicated in Mendelian neuropsychiatric syndromes. *Psychiatry Clin Neurosci* **73**, 11-19, doi:10.1111/pcn.12788 (2019).

22 Urresti, J. *et al.* Cortical organoids model early brain development disrupted by 16p11.2 copy number variants in autism. *Mol Psychiatry*, doi:10.1038/s41380-021-01243-6 (2021).

23 Mei, L. & Nave, K. A. Neuregulin-ERBB signaling in the nervous system and neuropsychiatric diseases. *Neuron* **83**, 27-49, doi:10.1016/j.neuron.2014.06.007 (2014).

24 Boldrini, M. *et al.* Hippocampal angiogenesis and progenitor cell proliferation are increased with antidepressant use in major depression. *Biol Psychiatry* **72**, 562-571, doi:10.1016/j.biopsych.2012.04.024 (2012).

25 Reif, A. *et al.* Neural stem cell proliferation is decreased in schizophrenia, but not in depression. *Mol Psychiatry* **11**, 514-522, doi:10.1038/sj.mp.4001791 (2006).

26 Patricio, P., Mateus-Pinheiro, A., Sousa, N. & Pinto, L. Re-cycling paradigms: cell cycle regulation in adult hippocampal neurogenesis and implications for depression. *Mol Neurobiol* **48**, 84-96, doi:10.1007/s12035-013-8422-x (2013).

27 Cunningham, K. A. & Watson, C. S. Cell cycle regulation, neurogenesis, and depression. *Proc Natl Acad Sci U S A* **105**, 2259-2260, doi:10.1073/pnas.0800029105 (2008).

28 Licht, T. & Keshet, E. The vascular niche in adult neurogenesis. *Mech Dev* **138 Pt 1**, 56-62, doi:10.1016/j.mod.2015.06.001 (2015).

29 Byers, A. L. & Yaffe, K. Depression and risk of developing dementia. *Nat Rev Neurol* **7**, 323-331, doi:10.1038/nrneurol.2011.60 (2011).

30 Van Der Veen, F. M., Evers, E. A. T., Deutz, N. E. P. & Schmitt, J. A. J. Effects of Acute Tryptophan Depletion on Mood and Facial Emotion Perception Related Brain Activation and Performance in Healthy Women with and without a Family History of Depression. *Neuropsychopharmacol* **32**, 216-224, doi:10.1038/sj.npp.1301212 (2007).

31 Jenkins, T., Nguyen, J., Polglaze, K. & Bertrand, P. Influence of Tryptophan and Serotonin on Mood and Cognition with a Possible Role of the Gut-Brain Axis. *Nutrients* **8**, 56, doi:10.3390/nu8010056 (2016).

32 Van der Does, A. J. The effects of tryptophan depletion on mood and psychiatric symptoms. *J Affect Disord* **64**, 107-119, doi:10.1016/s0165-0327(00)00209-3 (2001).

33 Beacher, F. D. *et al.* Acute tryptophan depletion attenuates conscious appraisal of social emotional signals in healthy female volunteers. *Psychopharmacology (Berl)* **213**, 603-613, doi:10.1007/s00213-010-1897-5 (2011).

34 Biskup, C. S. *et al.* Effects of acute tryptophan depletion on brain serotonin function and concentrations of dopamine and norepinephrine in C57BL/6J and BALB/cJ mice. *PLoS ONE* **7**, e35916, doi:10.1371/journal.pone.0035916 (2012).

35 Deza-Araujo, Y. I. *et al.* Acute tryptophan loading decreases functional connectivity between the default mode network and emotion-related brain regions. *Hum Brain Mapp* **40**, 1844-1855, doi:10.1002/hbm.24494 (2019).
